# Polyphasic characterization of *Biscogniauxia papillata* sp. nov. (*Graphostromataceae*) and isolation of the cytotoxic cyclic pentapeptide cyclobiscognioxin A

**DOI:** 10.1007/s11557-025-02114-y

**Published:** 2026-01-08

**Authors:** Sarunyou Wongkanoun, Esteban Charria-Girón, Boonchuai Chainuwong, Prasert Srikitikulchai, Natapol Pornputtapong, Jennifer Luangsa-ard, Sherif S. Ebada, Marc Stadler

**Affiliations:** 1 Department of Biochemistry and Microbiology, Center of Excellence for DNA Barcoding of Thai Medicinal Plants, Faculty of Pharmaceutical Sciences, Chulalongkorn University, Bangkok, 10330 Thailand; 2 Plant Microbe Interaction Research Team (APMT), National Center for Genetic Engineering and Biotechnology (BIOTEC), 113 Thailand Science Park, Phahonyothin Road, Khlong Nueng, Khlong Luang, Pathum Thani, 12120 Thailand; 3 Department of Microbial Drugs, Helmholtz Centre for Infection Research GmbH (HZI), German Centre for Infection Research Association (DZIF), Partner Site Hannover-Braunschweig, Inhoffenstraße 7, Brunswick, 38124 Germany; 4 Bioinformatics Group, Wageningen University & Research, Droevendaalsesteeg 1, 6708 PB Wageningen, the Netherlands; 5 National Biobank of Thailand (NBT), National Center for Genetic Engineering and Biotechnology (BIOTEC), 111 Thailand Science Park, Phahonyothin Road, Khlong Nueng, Khlong Luang, Pathum Thani, 12120 Thailand; 6 Department of Pharmacognosy, Faculty of Pharmacy, Ain Shams University, Cairo, 11566 Egypt; 7 Institute of Microbiology, Technische Universität Braunschweig, Spielmannstraße 7, 38106 Brunswick, Germany

**Keywords:** Phylogeny, Cyclic peptides, Secondary metabolites, *Sordariomycetes*, *Xylariales*

## Abstract

The *Graphostromataceae* (*Xylariales*) mainly contains species known for their endophytic lifestyle, although some may shift to opportunistic pathogenicity under host stress and subsequently colonize woody tissues as saprotrophs. The largest genus, *Biscogniauxia*, remains largely unexplored and would greatly benefit from a polyphasic taxonomic revision. Likewise, the secondary metabolism of these fungi has been only partially studied compared to related families within the *Xylariales*, despite their ecological versatility and potential latent pathogenicity. Herein, we introduce *Biscogniauxia papillata* as a new species from Thailand, based on morphological features and robust multi-locus phylogenetic analyses. Secondary metabolite profiling in different media, followed by preparative chromatography, led to the isolation of a cyclic pentapeptide named cyclobiscognioxin A (**1**), along with a mellein derivative among its main components. Preliminary biological testing revealed that **1** exhibits potent cytotoxic properties but lacks antimicrobial properties in our assays. The combined morphological and molecular phylogenetic evidence unequivocally distinguishes *B. papillata* from other members of the genus, confirming its status as a new species. Moreover, our chemical investigation highlights the underexplored biosynthetic potential of *Biscogniauxia* and expands the known chemical diversity within the genus, which might contribute to their opportunistic lifestyle.

**Supplementary Information:**

The online version contains supplementary material available at 10.1007/s11557-025-02114-y.

## Introduction

The taxonomic revision of *Biscogniauxia* by Ju et al. (1998) recognized 49 taxa within the genus. Since then, the number of accepted species has significantly increased, with 118 taxa currently listed worldwide Robert et al. (2005) www.mycobank.org. Morphologically, *Biscogniauxia* is characterized by widely effuse stromata with separate ostioles on the surface. The perithecia are primarily arranged in a single layer, though sometimes polystichous. Asci are cylindrical, 8-spored, and may or may not possess an amyloid apical apparatus while ascospores are predominantly uniseriate, rarely biseriate, ellipsoid, and brown, with or without germ slits (Ju et al. 1998; Vasilyeva et al. 2007).

The phylogenetic relationships of *Biscogniauxia* and its allied genera have been firmly established within the family *Graphostromataceae* M.E. Barr, J.D. Rogers and Y.M. Ju, and refined by M. Stadler, L. Wendt and Sir based on multi-gene phylogenetic analyses and comprehensive macro- and microscopic features (Wendt et al. 2018; Samarakoon et al. 2022). This family includes *Graphostroma* Piroz., a genus characterized by its hyaline, elongated ascospores and bipartite stromata, as well as *Biscogniauxia*, *Camillea* Fr., *Cryptostroma* P.H. Greg. and S. Waller, *Obolarina* Pouzar, and *Vivantia* J.D. Rogers, Y.M. Ju and Cand. These morphological similarities further support their classification within *Graphostromataceae* (Wendt et al. 2018; Daranagama et al. 2018). These taxa display significant ecological versatility, acting as both saprotrophs and opportunistic pathogens depending on environmental conditions. This dual lifestyle underscores the ecological role of *Biscogniauxia* spp. in forest ecosystems, where it contributes to wood decomposition as well as plant-pathogen interactions (Ju et al. 1998).

While recent studies have started to delve into the translational potential of the ecological versatility of *Graphostromataceae*, particularly regarding their biotechnological relevance and secondary metabolism, the actual ecological functions of tthe molecules they produce remain poorly understood, especially in the context of plant diseases (Helaly et al. 2018; Purbaya et al. 2023). For instance, the phytotoxic properties of certain metabolites, such as biscopyran, suggest a possible role in plant–pathogen interactions and may contribute to the virulence mechanisms of *Biscogniauxia* species (Evidente et al. 2005). However, our understanding of the pathogenic traits in these fungi is hindered by the lack of mechanistic studies on their secondary metabolites, uncertainty about the production of these compounds across related taxa, and the absence of evidence for their involvement during infection.

As a part of our taxonomic studies on xylarialean fungi in Thailand, supported by extensive fieldwork, we identified a novel species of *Biscogniauxia*. This study is dedicated to its comprehensive taxonomic description, incorporating detailed macro- and microscopic features, accompanied by illustrations. Additionally, we evaluated its secondary metabolite production under different conditions, characterized its main components after preparative isolation and structure elucidation, and assessed their antimicrobial and cytotoxic properties.

## Materials and methods

### Sample collection and cultivation

The specimens in this study were collected from the community forests in northern Thailand. Pictures of the specimens in their natural habitat were captured using a Canon 60D digital camera (Canon, Tokyo, Japan). Pure isolates were obtained by culturing on potato dextrose agar (PDA) using the multiple spore isolation technique on the same day as field collection. After a few days of incubation, hyphal tips were excised and transferred to fresh agar plates. Pure cultures were preserved in the BIOTEC Culture Collection (BCC) and the National Biobank of Thailand (NBT), while the dried voucher specimens were deposited in the BIOTEC Bangkok Herbarium (BBH), Thailand, respectively.

### Morphological characterization and temperature growth profile

Morphological features, such as stromatal size and shape, perithecia, asci, apical apparatus, and ascospores were examined in accordance with Ju et al. (1998) using a Nikon (Bangkok, Thailand) Eclipse Ni connected with a Nikon microscope camera DS-Ri2 and a stereo dissecting microscope Nikon SMZ18 (Bangkok, Thailand). The abbreviations used in the descriptions were as follows: *x̄* = mean of the measurements per specimen, and *n* = number of measurements per specimen. Fungal cultures were grown on several media, i.e., oatmeal agar (Difco OA; Becton Dickinson, Carlsbad, CA, USA); potato dextrose agar (Difco PDA); yeast malt glucose agar (1% malt extract, 0.4% glucose, and 0.4% yeast extract; agar 1%; YMGA); and malt extract agar (2% malt extract, 1% glucose, and 0.1% peptone; agar 1%; MEA). The strains were inoculated in one point on 90 mm Petri dishes and incubated at 25 °C in darkness. Conidiogenous cells and conidiophore branching patterns of the anamorph were examined as proposed by Ju and Rogers (1998). Furthermore, the cultures colors were documented following the color chart of Rayner (1970). Cardinal temperatures were determined for *Biscogniauxia papillate*. The strains were grown on MEA, OA, PDA, and YMGA for 14 d at 5, 25, and 35 °C in darkness.

### DNA extraction, polymerase chain reaction (PCR), and sequencing

Genomic DNA from fungal mycelia was isolated according to the methods of O’Donnell and Cigelnek (1997) and Sakayaroj et al. (2011). The internal transcribed spacer regions (ITS), large subunit of the rDNA (LSU), RNA polymerase II (*RPB2*), and beta tubulin (*TUB2*) were amplified using standard primers introduced by White et al. (1990; ITS1, ITS4, and ITS5); Vilgalys and Hester (1990; LR5 and LROR); Liu et al. (1999: *RPB2*–5F and 7Cr); and O’Donnell and Cigelnik (1997; T1 and T22). PCR was conducted in 25 µL reaction volume consisting of 1 × PCR buffer, 200 µM of each of the four dNTPs, 2.5 mM MgCl_2_, 1 U Taq DNA polymerase recombinant (Thermo Scientific, USA), 0.5 µM of each primer, and 50–100 ng of DNA template. Amplification was performed using a T100TM thermal cycler (BIO-RAD Laboratories, Inc., California) under the following conditions: 94 °C for 2 min, followed by 35 cycles of denaturation at 94 °C for 1 min, annealing at a suitable temperature for 1 min, extension at 72 °C for 2 min, and a final extension of 72 °C for 10 min. The annealing temperatures were 55 °C for ITS and LSU, 54 °C for *RPB2*, and 53 °C for *TUB2*. PCR products were sent to Macrogen Co. (Seoul, Korea) for purification and sequencing using the same primers as in the PCR amplification. DNA sequences were checked and assembled using BioEdit v. 7.2.5 (Hall 1999) and AliView v. 1.28 (Larsson, A. 2014). All newly generated sequences were submitted to GenBank (https://www.ncbi.nlm.nih.gov/) and listed in Table 1.

**Table 1:** Taxa used in the phylogenetic analyses and their corresponding GenBank accession numbers

Species	Strain number	Status	GenBank accession numbers				References
			**ITS**	***RPB2***	***TUB2***	**LSU**	
*Amphirosellinia fushanensis*	HAST 91111209	holotype	GU339496	GQ848339	GQ495950	N/A	Hsieh et al. (2010)
*Amphirosellinia nigrospora*	HAST 91092308	holotype	GU322457	GQ848340	GQ495951	N/A	Hsieh et al. (2010)
*Astrocystis concavispora*	MFLUCC 14–0174	isotype	KP297404	KP340532	KP406615	KP340545	Daranagama et al. (2015)
*Barrmaelia oxyacanthae*	CBS 142770		MF488988	MF488997	MF489016	N/A	Voglmayr et al. (2018)
*Barrmaelia rhamnicola*	CBS 142772	epitype	MF488990	MF488999	MF489018	N/A	Voglmayr et al. (2018)
*Biscogniauxia anceps*	YMJ 123	authority	EF026132	JX507777	AY951671	N/A	Hsieh et al. (2010)
*Biscogniauxia arima*	YMJ 122	Isotype	EF026150	GQ304736	AY951672	N/A	Hsieh et al. (2010)
*Biscogniauxia capnodes*	YMJ 138	authority	EF026131	JX507779	AY951675	N/A	Hsieh et al. (2010)
*Biscogniauxia citriformis*	YMJ129	authority	JX507801	JX507781	AY951678	N/A	Mirabolfathy et al. (2013)
*Biscogniauxia citriformis*	YMJ88113012	authority	JX507800	JX507780	AY951677	N/A	Mirabolfathy et al. (2013)
*Biscogniauxia cylindrispora*	YMJ 89092701	authority	EF026133	JX507782	AY951679	N/A	Hsieh et al. (2010)
*Biscogniauxia formosana*	YMJ89032201	authority	JX507802	JX507783	AY951680	N/A	Mirabolfathy et al. (2013)
*Biscogniauxia glaucae*	GMBC0007	Holotype	MT624046	MT622652	MT622654	N/A	Li et al. (2021))
*Biscogniauxia glaucae*	GMBC0029		MT624047	MT622653	MT622655	N/A	Li et al. (2021))
*Biscogniauxia granmoi*	YMJ 135	authority	JX507803	JX507784	AY951681	N/A	Mirabolfathy et al. (2013)
*Biscogniauxia latirima*	YMJ 90080703	authority	EF026135	JX507786	AY951683	N/A	Mirabolfathy et al. (2013)
*Biscogniauxia latirima*	YMJ89101101	authority	JX507804	JX507785	AY951682	N/A	Mirabolfathy et al. (2013)
*Biscogniauxia marginata*	CBS 124505	-	KU684016	KU684310	KU684124	N/A	U’Ren et al. (2016)
*Biscogniauxia mediterranea*	YMJ 147	authority	EF026134	GQ844765	AY951684	N/A	Hsieh et al. (2010)
*Biscogniauxia mediterranea*	AZ0703	-	HM123416	KU684217	KU684122	N/A	U’Ren et al. (2016)
*Biscogniauxia nummularia*	CBS 969.70	-	MH860015	KU684281	KU684125	N/A	Vu et al. (2019)
*Biscogniauxia nummularia*	MUCL 51395	epitype	NR_153649	KY624236	KX271241	KT281894	Wendt et al. (2018)
***Biscogniauxia papillata***	**BCC 36828**	**holotype**	**PQ586363**	**PQ604605**	N/A	**PQ586365**	**This study**
***Biscogniauxia papillata***	**BCC 83050**	**authority**	**PQ586364**	**PQ604606**	N/A	**PQ586366**	**This study**
*Biscogniauxia petrensis*	LC5697	-	KU746669	KY883231	KU746761	KU746715	Zhang et al. (2017)
*Biscogniauxia philippinensis* var. *microspora*	YMJ 89041101	authority	EF026136	JX507787	AY951685	N/A	Hsieh et al. (2010)
*Biscogniauxia repanda*	ATCC 62606	-	KY610383	KY624237	KX271242	KY610428.	Wendt et al. (2018)
*Biscogniauxia rosacearum*	Bx26	type	KT253493	N/A	KT253527	N/A	Raimondo et al. (2016)
*Biscogniauxia simplicior*	YMJ 136	authority	EF026130	JX507788	AY951686	N/A	Hsieh et al. (2010)
*Biscogniauxia uniapiculata*	YMJ90080608	authority	JX507805	JX507789	AY951687	N/A	Mirabolfathy et al. (2013)
*Camillea broomeana*	GMB0218		MW854657	GMB0218	MW855491	MW854663.	Li et al. (2021))
*Camillea obularia*	ATCC_28093	-	KY610384	KY624238	KX271243	KY610429	Wendt et al. (2018)
*Camillea* sp.	MFLU 18–0786	-	MW240614	MW342618	N/A	MN244210	Samarakoon,M.C submitted directly.
*Camillea tinctor*	YMJ363	authority	JX507806	JX507790	JX507795	OQ871479	Hsieh and Ju (direct submission)
*Camillea tinctor*	CBS 203.56	-	KU683753	KU684282	N/A	N/A	U’Ren et al. (2016)
*Clypeosphaeria mamillana*	CBS 140735	epitype	KT949897	MF489001	MH704637	MH554225	Jaklitsch and Gardiennet (2016)
*Collodiscula bambusae*	GZU H0102	holotype	KP054279	KP276675	KP276674	KP054280	Li et al. (2015)
*Collodiscula japonica*	CBS 124266	-	JF440974	KY624273	KY624316	MH874889	Jaklitsch and Voglmayr (2012; ITS, LSU), Wendt et al. (2018; *RPB2*,*TUB2*)
*Cryptostroma corticale*	Acer7	-	HG934114	HG934119	HG934105	N/A	Koukol et al. (2015)
*Cryptostroma corticale*	CBS 216.52	-	MH857008	HG934116	HG934102	N/A	Vu et al. (2019)
*Cryptostroma corticale*	CBS 217.52	-	HG934111	HG934117	HG934103	N/A	Koukol et al. (2015)
*Cryptostroma corticale*	CBS 218.52	-	HG934112	HG934118	HG934104	N/A	Koukol et al. (2015)
*Entosordaria perfidiosa*	CBS 142773	epitype	MF488993	MF489003	MF489021	N/A	Voglmayr et al. (2018)
*Entosordaria quercina*	CBS 142774	holotype	MF488994	MF489004	MF489022	N/A	Voglmayr et al. (2018)
*Graphostroma guizhouense*	GMBC0219	holotype	MW854659	MW855487	MW855490	MW854664	Li et al. (2021)
*Graphostroma guizhouense*	GMBC0008	-	MW854658	MW855486	MW855489	MW854662	Li et al. (2021)
*Graphostroma platystomum*	CBS 270.87	type	JX658535	DQ836893	HG934108	DQ836906	Stadler et al. (2014; ITS), Zhang et al. (2017; LSU), Koukol et al. (2015; *TUB2*), Wendt et al. (2018; *RPB2*)
*Graphostroma platystomum*	CBS:146066	-	MT223799	MT223680	MT223734	N/A	Hsieh et al. (2010)
*Hypocreodendron sanguineum*	J.D.R. 169	-	GU322433	GQ844819	GQ487710	N/A	Hsieh et al. (2010)
*Hypoxylon pulicicidum*	MUCL49879	holotype	JX183075	KY624280	JX183072	KY610492.	Bills et al. (2012; ITS, TUB2), Wendt et al.(2018; LSU, RPB2)
*Hypoxylon rickii*	MUCL 53309	epitype	KC968932	KY624281	KC977288	N/A	Kuhnert et al. (2014; ITS, *TUB2*), Wendt et al.(2018; LSU, *RPB2*)
*Kretzschmaria deusta*	CBS 163.93	holotype	KC477237	KY624227	KX271251	KY610458	Stadler et al. (2013, ITS), Wendt et al. (2018, LSU, RPB2, *TUB2*)
*Linosporopsis ischnotheca*	LIF1	epitype	MN818952	MN820708	MN820715	N/A	Voglmayr and Beenken (2020)
*Linosporopsis ochracea*	LIO3	epitype	MN818958	MN820714	MN820721	N/A	Voglmayr and Beenken (2020)
*Nemania bipapillata*	HAST 90080610	-	GU292818	GQ844771	GQ470221	N/A	Hsieh et al. (2010)
*Nemania primolutea*	HAST 91102001	holotype	EF026121	GQ844767	EF025607	N/A	Hsieh et al. (2010)
*Obolarina dryophila*	MUCL 49882	-	GQ428316	KY624284	GQ428321	GQ428316	Pažoutová et al. (2010); Wendt et al. (2018; *RBB2*)
*Obolarina dryophila*	H76	-	GQ428317	N/A	GQ428323	N/A	Pažoutová et al. (2010)
*Obolarina* sp.	YMJ 1461	-	JX507807	JX507792	JX507796	N/A	Mirabolfathy M et al. (2013)
*Podosordaria mexicana*	WSP 176	-	GU324762	GQ853039	GQ844840	N/A	Hsieh et al. (2010)
*Poronia pileiformis*	WSP 88113001	epitype	GU324760	GQ853037	GQ502720	N/A	Hsieh et al. (2010)
*Rosellinia aquila*	MUCL 51703	-	KY610392	KY624285	KX271253	KY610460	Wendt et al. (2018)
*Dematophora buxi*	J.D.R. 99	-	GU300070	GQ844780	GQ470228	N/A	Hsieh et al. (2010)
*Stilbohypoxylon quisquiliarum*	Y.M.J. 172	-	EF026119	GQ853020	EF025605	N/A	Hsieh et al. (2010)
*Xylaria adscendens*	J.D.R. 865	-	GU322432	GQ844818	GQ487709	N/A	Hsieh et al. (2010)
*Xylaria bambusicola*	WSP 205	holotype	EF026123	GQ844802	AY951762	N/A	Hsieh et al. (2010)
*Sarcoxylon compunctum*	CBS 359.61	-	KT281903	KY624230	KX271255	KY610462	Senanayake et al. (2015; ITS), Wendt et al.(2018; LSU, *RPB2*, *TUB2*)
*Xylaria hypoxylon*	CBS 122620	-	KY610407	KY624231	KX271279	KY610495	Sir et al. (2016; *TUB2*), Wendt et al. (2018; ITS, LSU, *RPB2*)
*Xylaria hypoxylon*	95082001 (HAST)	-	GU300095	GQ844811	GQ487703	N/A	Hsieh et al. (2010)

New taxa proposed in this study are in bold.

### Molecular phylogenetic inference

All sequences were aligned using Multiple Sequence Comparison by Log-Expectation (MUSCLE) (Edgar 2004) and manually refined. Multiple sequence alignments were analyzed together with closely matched sequences and other reference taxa obtained from GenBank as shown in Table 1. Phylogenetic analyses were performed using maximum likelihood (ML) and Bayesian algorithm (MB). The ML tree and bootstrap analyses were conducted through the CIPRES Science Gateway V. 3.3 (Miller et al. 2010) using RAxML 8.2.4 (Stamatakis 2014) with the BFGS method to optimize GTR rate parameters. Bayesian posterior probabilities of the branches were estimated using MrBayes 3.0B4 (Huelsenbeck and Ronquist 2001) with the best-fit model (GTR + I + G) selected by AIC in Mr Modeltest 2.2 (Nylander 2004) and tested using hierarchical likelihood ratio tests (hLRTs). Three million generations were run in four Markov chains, sampling every 100 generations, with a burn-in value set at 5000 sampled trees. Sequences of *Hypoxylon pulicicidum* (MUCL49879) and *Hypoxylon rickii* (MUCL 53309) were used as outgroups.

**Table 2:** Comparison of morphological features among *Biscogniauxia* species closely related to *B. papillata* and other similar species

Taxa	Ascospore	Perithecia	Ostioles	Anamorph
*B. capnodes* (Berk.) Y.M. Ju and J.D. Rogers, Mycotaxon 66: 23. 1998.	8.5–15 × 5–7.5 µm	Obovoid to tubular, 0.4–0.8 mm high., 0.2–0.4 mm diam	Punctate and usually surrounded by slightly raised rim, sometimes overlain with white substance.	Periconiella-like
B. papillata*	**(12–) 13–15 (–16) × (7–) 8–9**	**Numerous individuals arranged in rosettes and discharging through a single ostiolar 0.6–1 mm high, 0.2–0.3 mm**	**Papillate, without surrounded by a slightly raised rim**	**Periconiella-like**
*B. plana (*Petch) Y.M. Ju and J.D. Rogers, Mycotaxon 66: 48. 1998.	11–14 × 5.5–7.5	Numerous individuals arranged in rosettes and discharging through a single ostiolar 0.3–0.5 mm high, 0.05 (–0.1) mm diam	Slightly lower than to slightly higher than stromatal surface, with openings punctate or slightly papillate	Periconiella-like
*B.communapertura* Y.M. Ju and J.D. Rogers, Mycotaxon 66: 31. 1998	8–11.5 × 4.5–6	Numerous individuals arranged in rosettes and discharging through a single ostiolar canal, 0.3–0.5 high, 0.1–0.2 diam	Widely spaced, higher than stromatal surface, with openings coarsely papillate	N/a
*B. capnodes var. limonispora* Y.M. Ju and J.D. Rogers, Mycotaxon 66: 26. 1998.	11.5–14 × 6–7.5	Obovoid to tubular, 0.4–0.8 mm high, 0.2–0.4 mm diam	Punctate and usually surrounded by slightly raised rim, sometimes overlain with white substance	Periconiella-like
*B. capnodes var. limonispora* Y.M. Ju and J.D. Rogers Mycotaxon 66: 26. 1998. Specimens reported from Martinique (French West Indies) Fournier et al. (2017)	(11.7–) 12.8–14.5 (–15) × (7.3–) 7.7–8.9 (–9.3)	Individual ostioles, two adjacent perithecia at times sharing a common ostiole. 0.3–0.5 mm high × 0.1–0.25 diam	Punctate, surrounded by a slightly raised rim, 25–70 µm diam, inconspicuous, evenly distributed, at times plugged with greyish to whitish substance	Periconiella-like
*B. nummularia* (Bull.: Fr.) Kuntze, Revis. Gen. Pl. 2: 398. 1891.	10–13 (–14) × (6.5–) 7.5–8.5	Obovoid, 0.3–0.5 mm diam, 0.5–0.7 mm high.	Higher than stromatal surface with openings slightly papillate, or lower than stromatal surface with openings punctate and usually surrounded by slightly raised rim	Periconiella-like
*B. anceps* (Sacc.) J.D. Rogers, Y.M. Ju and Cand., Mycol. Res. 100: 669. 1996.	(13–) 14–18 (–19) µm total length × 7–9 (–10) µm broad at the broadest part, the larger cell 10–13 µm long and the smaller cell 5–7 µm long × 4–7 µm broad.	0.2–0.4 mm diam	Umbilicate, located in grey depressed areas	Periconiella-like

*This study

### General analytical procedures

Electrospray mass (ESI–MS) spectra were recorded with an UltiMate® 3000 Series UHPLC system (Thermo Fisher Scientific, Waltham, MA, USA) connected to an amazon speed® ESI-Ion Trap-MS (Bruker, Billerica, MA, USA) mass spectrometer. A C_18_ Acquity® UPLC BEH column (2.1 × 50 mm, 1.7 µm; Waters, Milford, MA, USA) was used as stationary phase. HPLC parameters were set as follows: solvent A: H_2_O + 0.1% formic acid, solvent B: acetonitrile (MeCN) + 0.1% formic acid; gradient 5% B for 0.5 min, increasing to 100% B over 19.5 min, followed by 5 min at 100% B; flow rate 0.6 mL/min, with diode array (DAD) detection in the range of 200–600 nm.

High-resolution electrospray ionization mass spectrometry (HR-ESI–MS) spectra were obtained using an Agilent 1200 Infinity Series HPLC (Agilent Technologies, Santa Clara, CA, USA) connected to a maXis® Electrospray Time-of-flight mass spectrometer (ESI-TOF–MS; Bruker). The HPLC conditions were the same as for ESI–MS measurements. NMR spectra were recorded with an Avance III 600 spectrometer (Bruker, ^1^H NMR: 600 MHz, ^13^C NMR: 150 MHz) in deuterated methanol. Optical rotation was measured with a MCP 100 circular polarimeter (Anton Paar, Graz, Austria), and UV/Vis spectra were acquired using a UV-2450 spectrophotometer (Shimadzu, Kyoto, Japan). Electronic circular dichroism (ECD) spectra were acquired using a J-815 spectropolarimeter (JASCO, Pfungstadt, Germany).

### Fungal cultivation and isolation of secondary metabolites

For scaled-up cultivation of BCC 36828, this fungus was first grown in YM agar at 23 °C. The seed culture was done in 250-mL flasks each containing 50 mL semi-viscous SMYA medium (Serrano et al. 2017) (maltose 40 g/L, yeast extract 10 g/L, meat peptone 10 g/L, agar 4 g/L). Subsequently, inoculation was done by adding five pieces (ca. 25 mm 2 each) of a well-grown agar-plate to each flask. Seed cultures were incubated for 5 days on a shaker (23 °C, 230 rpm). Afterwards, 6 mL of the seed culture were transferred into the new 500-mL conical flasks, containing the solid rice culture medium (28 g brown rice and 100 mL of base liquid medium). The base liquid medium consisted of yeast extract (1 g/L), di-sodium tartrate dihydrate (0.5 g/L), and KH₂PO₄ (0.5 g/L). Finally, solid cultures were incubated for 15 days in the dark at 23 °C without shaking.

For secondary metabolite extraction, the mycelia on the rice were covered with acetone and sonicated for 30 min at 40 °C. The acetone extract was separated from the mycelia using cellulose filter paper (MN 615 1/4 Ø 185 mm, Macherey–Nagel GmbH & Co. KG, Düren, Germany). The extraction and filtration steps were repeated twice, and the obtained acetone phases were combined and evaporated under reduced pressure at 40 °C (evaporator: Heidolph Instruments GmbH & Co. KG, Germany; pump: Vacuubrand GmbH & Co. KG, Wertheim am Main, Germany) to obtain an aqueous residue. The remaining aqueous residue was dispersed in 1 L of H_2_O and extracted twice, using 1 L of ethyl acetate. The ethyl acetate phases were combined and evaporated to dryness under reduced pressure at 40 °C to yield 547.33 mg of the crude extract.

For the isolation of cyclobiscognioxin A (**1**), the obtained crude extract obtained was portioned to 2 × 270 mg and purified using a PLC 2250 preparative HPLC system (Gilson, Middleton, WI, USA) with a Gemini C_18_ (50 × 250 mm, 10 µm; Phenomenex®, Torrance, CA, USA as stationary phase. The HPLC conditions were as follows: solvent A: H_2_O + 0.1% formic acid, solvent B: MeCN + 0.1% formic acid; flow rate: 50 mL/min, gradient: isocratic conditions at 5% B for 5 min, followed by an increase to 55% B in 50 min, then increasing to 100% B in 5 min, and ending with isocratic conditions at 100% B for 10 min. This yielded **1** (1.73 mg, *t*_R_ = 18 min) and **2** (0.9 mg, *t*_R_ = 38 min).

Cyclobiscognioxin A (1): White powder; UV–Vis (MeOH): λ_max_ 230 nm; HR-ESI–MS: *m/z* 600.4118 [M + H]^+^ (calcd. 600.4125 for C_33_H_54_N_5_O_5_^+^), 622.3948 [M + Na]^+^ (calcd. 622.3944 for C_33_H_53_N_5_NaO_5_^+^); ^13^C/^1^H NMR data (methanol-*d*_4_, 150 and 600 MHz): see Table S1 and comparable to those reported in literature (Li et al. 2004); C_33_H_53_N_5_O_5_ (599.82 g mol^−1^).

3,5-Dimethyl-8-methoxy-3,4-dihydroisocoumarin (2): Colorless amorphous solid; UV–Vis (MeOH): λ_max_ 210, 247, 316 nm; HR-ESI–MS: *m/z* 207.1017 [M + H]^+^ (calcd. 207.1021 for C_12_H_15_O_3_^+^), 229.0837 [M + Na]^+^ (calcd. 229.0841 for C_12_H_14_NaO_3_^+^); ^1^H NMR data (methanol-*d*_4_, 600 MHz): comparable to those reported in literature (Kokubun et al. 2003); ^13^C NMR data (methanol-*d*_4_, 150 MHz): δ_C_ 161.5 (C-1), 158.6 (C-8), 140.3 (C-5), 135.3 (C-6), 125.9 (C-4a), 113.2 (C-8a), 110.6 (C-7), 72.8 (C-3), 55.5 (C-11), 32.1 (C-4), 20.2 (C-9), 17.8 (C-10); C_12_H_14_O_3_ (206.24 g mol^−1^).

### Biological activities

The evaluation of the antimicrobial and cytotoxic properties from the isolated compounds was performed following the methods reported by Charria-Girón et al. (2025).

## Results and discussion

### Taxonomy

***Biscogniauxia papillata*** Srikit., Wongkan., Charria-Girón and Luangsa-ard, sp. nov.

**Fig. 1:**
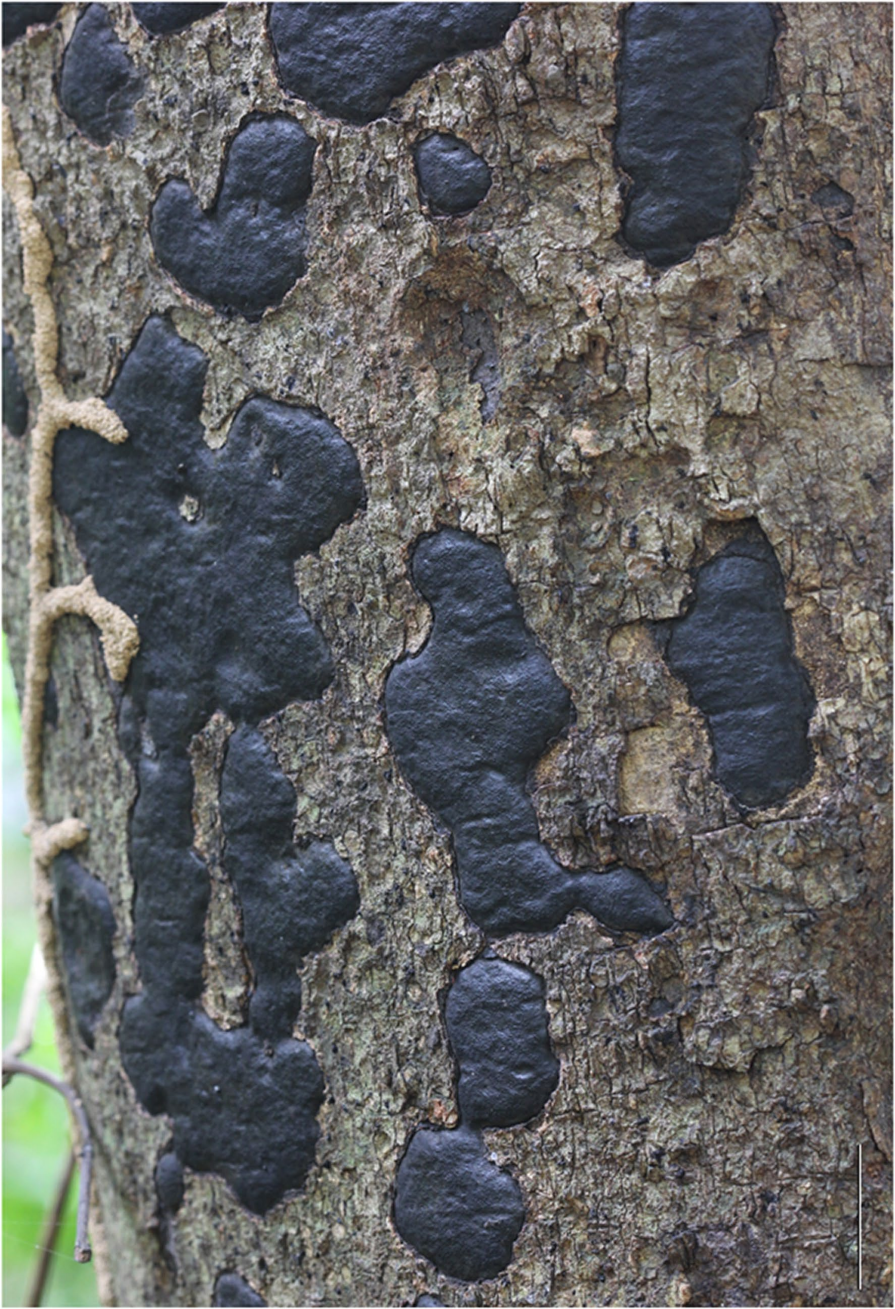
Stromata of *Biscogniauxia papillata* (BBH 25799) on the bark of an unidentified host tree. Stromata effused-pulvinate to irregular in outline, black, carbonaceous, solitary to coalescent, and partially embedded in the bark. Margins indistinct or lobed. Scale bar = 20 mm

**Fig. 2:**
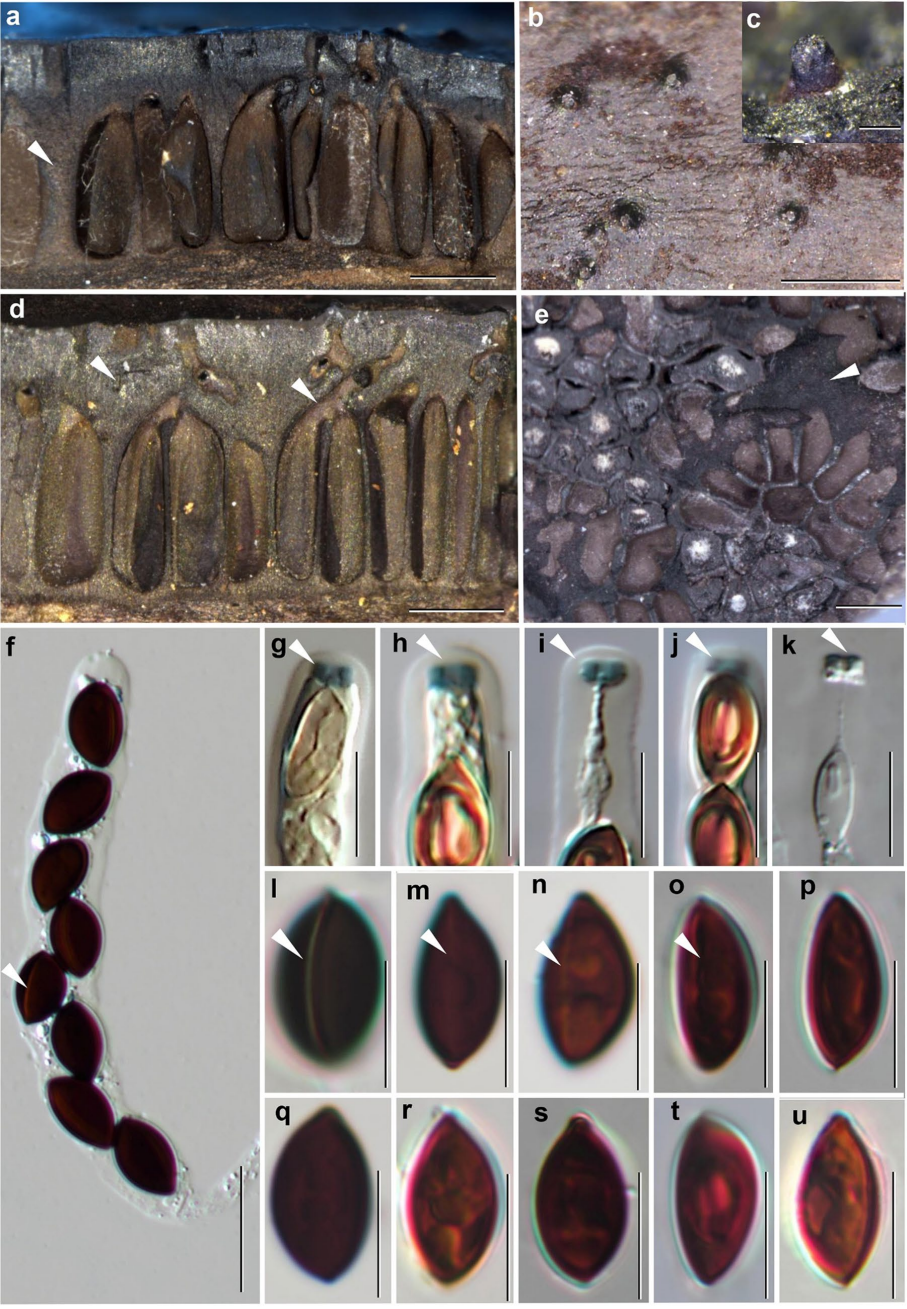
*Biscogniauxia papillata* (BBH 25799). **a**, **d** Stroma in longitudinal section showing the thick carbonaceous subsurface, the perithecia and the indistinct subperithecial tissue. **b** Shiny black papillate ostioles with truncate top. **c** Ostioles without the external surface. **e** Cross-section of the stroma showing rosette arrangement of perithecia and the presence of sterile tissue between them (white arrow). **f** Ascus. **g**–**j** Apical apparatus in Melzer’s reagent (white arrows). **k** Apical apparatus in 10% KOH reagent (white arrow). **l** Ascospore in 10% KOH reagent, showing conspicuous germ slits (white arrow). **m**–**o** Ascospores in distilled water, showing germ slits (white arrows). **p**–**u** Ascospores in distilled water. Scale bars: **a**–**b**,** d**–**e** 0.5 mm, **c** 100 µm, **f** 20 µm, **g**–**u** 10 µm

**Fig. 3:**
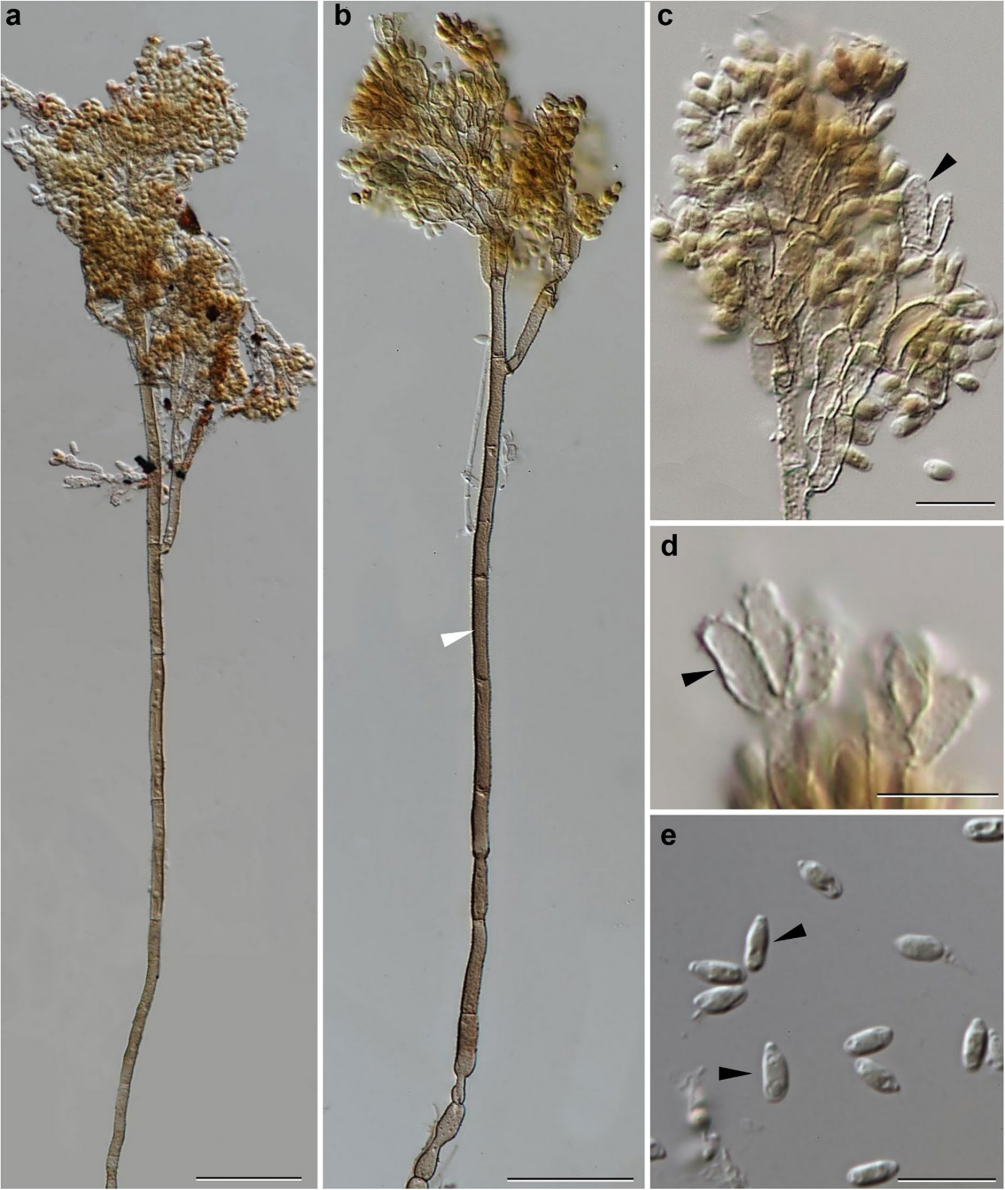
*Biscogniauxia papillata* (BCC 36828) **a**–**b** Conidiophores showing compact fertile head (arrow indicating melanized main axis) morph showing conidiophores with virgariella-like to nodulisporium-like branching patterns. **c** Fertile head with densely packed branchlets bearing conidiogenous cells (arrow). **d** Conidiogenous cells, cylindrical, hyaline, finely roughened, bearing inconspicuous collarettes (arrow). **e** Conidia, hyaline, smooth-walled, ellipsoid, often with flattened base (arrow). Scale bars: **a**–**b** = 40 µm; **c** = 20 µm; **d**–**e** = 10 µm

Figures 1, 2, and 3.

Mycobank MB 857465.

**Etymology**: “papillata,” referring to the ostioles of the new species having papillae or nipple-like or rounded projections on the stromatal surface*.*

**Diagnosis**: Differs from related *Biscogniauxia* species by having perithecia in clusters of 2–4 sharing a common ostiolar canal, papillate ostioles lacking a white rim, larger flask-shaped perithecia, and broadly ellipsoid, dark brown ascospores with a straight, full-length germ slit.

**Holotype**: Thailand: Chiang Rai Province, Phu Kaeng Waterfall, 19.443° N, 99.670° E, on decaying wood, 18 January 2012, P. Srikitikulchai, (BBH 25799), (ex-BCC 36828); DNA sequences of ex-holotype strain ITS– PQ586363, LSU– PQ586365, *RPB2*–PQ586365, *TUB2*–N/A.

**Teleomorph**: Stromata effused-pulvinate or irregular in shape, solitary to coalescent and distinctly bipartite, solitary to coalescent,10–120 mm long, 10–30 mm broad, 1.63–1.77 mm thick (Fig. 1); surface brown to blackish brown or black 0.035–0.071 mm thick (*x̅* = 0.048 mm; *n* = 10), carbonaceous forming a thin crust above perithecia layer 0.26–0.47 mm thick (*x̅* = 0.36 mm; *n* = 10) (Fig. 2a, d); the tissue between perithecia brownish, soft-textured; tissue beneath perithecia gray soft-textured and a blackish line separates the stromatal tissue from the host surface 0.14–0.35 mm, and the stromatal tissue is almost inseparable from the host substrate. Perithecia narrowly cylindrical to clavate, obpyriform to flask-shaped (lageniform) (0.76–)0.90–0.97(–1.01) × (0.18–)0.22–0.30(–0.44) mm (*x̅* = 0.94 × 0.28 mm; *n* = 25). Ostioles papillae or nipple-like or rounded projections on the stromatal surface (Fig. 2b–c). Asci cylindrical, the spore-bearing parts 82–89 × (6–)7–9 µm, the total length (86–)105–123 µm, with apical apparatus 4–5 × 1–2 µm (*x̅* = 4.48 × 2.02 µm; *n* = 10), bluing in Melzer’s reagent. Ascospores broadly ellipsoid, inequilateral, (12–)13–15(–16) × (7–)8–9 µm (*x̅* = 13.85 × 8.26 µm; *n* = 25), dark brown, smooth-walled, with a straight, full-length germ slit on the convex side.

**Culture characteristics**: Colonies on PDA, reaching the edge of the Petri dish in 1 week, azonate, at first whitish becoming Umber (9); reverse Umber (9) and Chestnut (40). Colonies on OA, reaching the edge of the Petri dish in 1 week, aerial mycelium at first whitish becoming Pale Olivaceous Grey (120) and Olivaceous Grey (121); reverse Olivaceous Grey (121). Colonies on YMGA reaching the edge of the Petri dish in 1 week, azonate, at first whitish becoming Umber (9); reverse Umber (9) and Chestnut (40). Colonies on MEA reaching the edge of the Petri dish in 1 week, azonate, at first whitish becoming Umber (9); reverse olivaceous (46) and Umber (9).

**Anamorph**: On OA conidiophores structure with periconiella-like to nodulisporium-like branching patterns, as described by Ju and Rogers (1996)

Conidiophores (Fig. 3a–b) erect, straight to slightly flexuous, unbranched along most of their length, 200–300 µm long, 5–6 µm wide, septate, with thickened walls; the main axis is melanized, brown to blackish brown, with a surface ranging from smooth to roughened. The fertile region is apical, composed of a dense cluster of short, closely packed branchlets bearing conidiogenous cells.

Conidiogenous cells (Fig. 3c–d) phialidic, cylindrical, hyaline, finely roughened, (7–)9.8–19.7 × 2.6–5.2 µm (*x̅* = 14.37 × 3.62 µm; *n* = 25), arranged in whorls or irregular clusters at the tips of branchlets; collarettes inconspicuous to short, flaring slightly. Conidia are produced holoblastically in a sympodial sequence from the apical region of each conidiogenous cell.

Conidia (Fig. 3e) hyaline, smooth-walled, aseptate, ellipsoid, (5–)5.8–6.9(–7.7) × 2.3–5.2 µm (*x̅* = 6.15 × 3.12 µm; *n* = 25), often with a flattened base marking the point of attachment to the conidiogenous cell; accumulating in slimy masses at the apex of the fertile head.

**Fig. 4:**
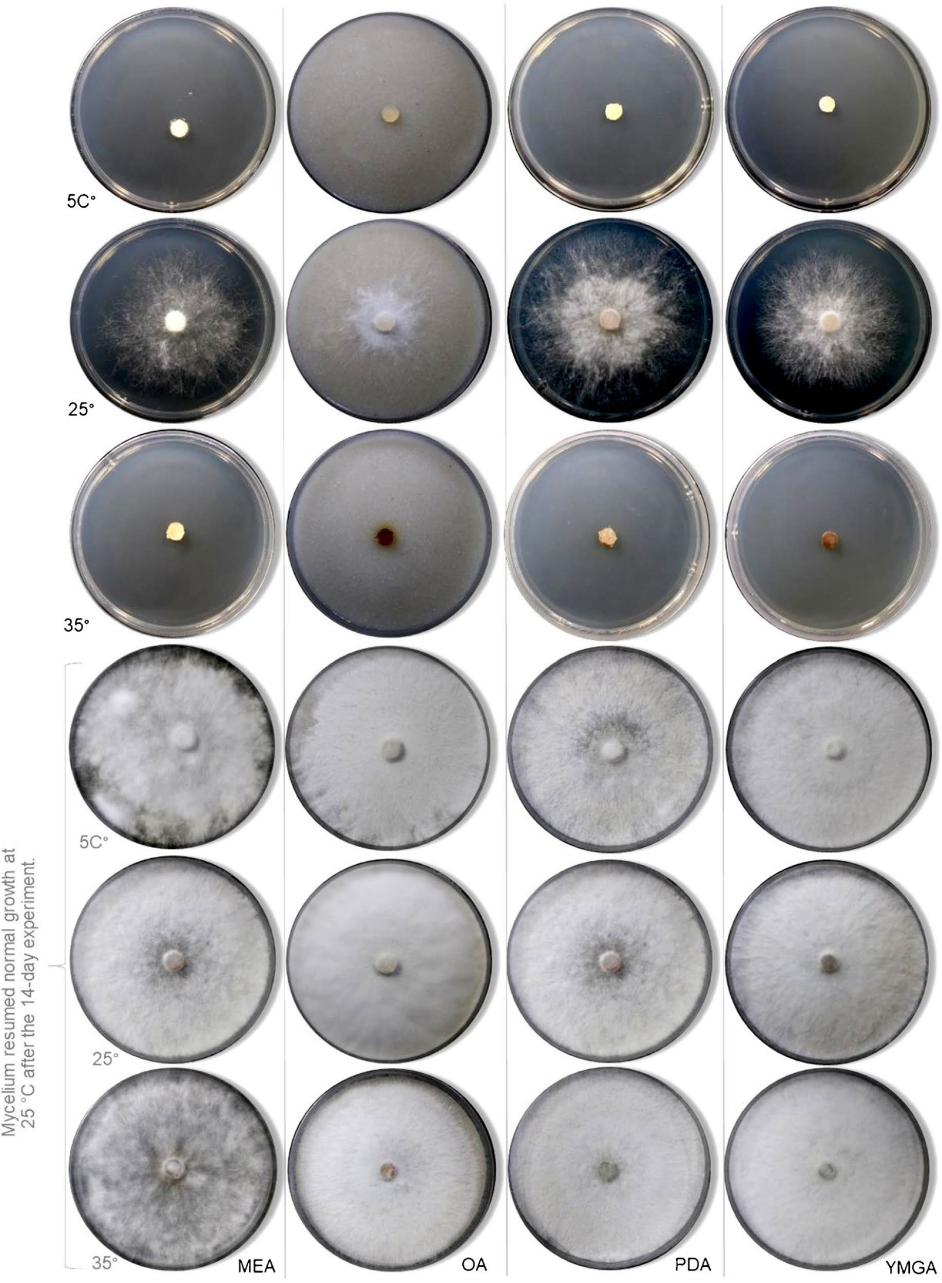
Temperature growth profile of the newly described species after 7 d of incubation on MEA, OA, PDA and YMGA at 5 °C, 25 °C, and 35 °C. Normal mycelial growth resumed at 25 °C after incubation under different temperatures 14 days

**Fig. 5:**
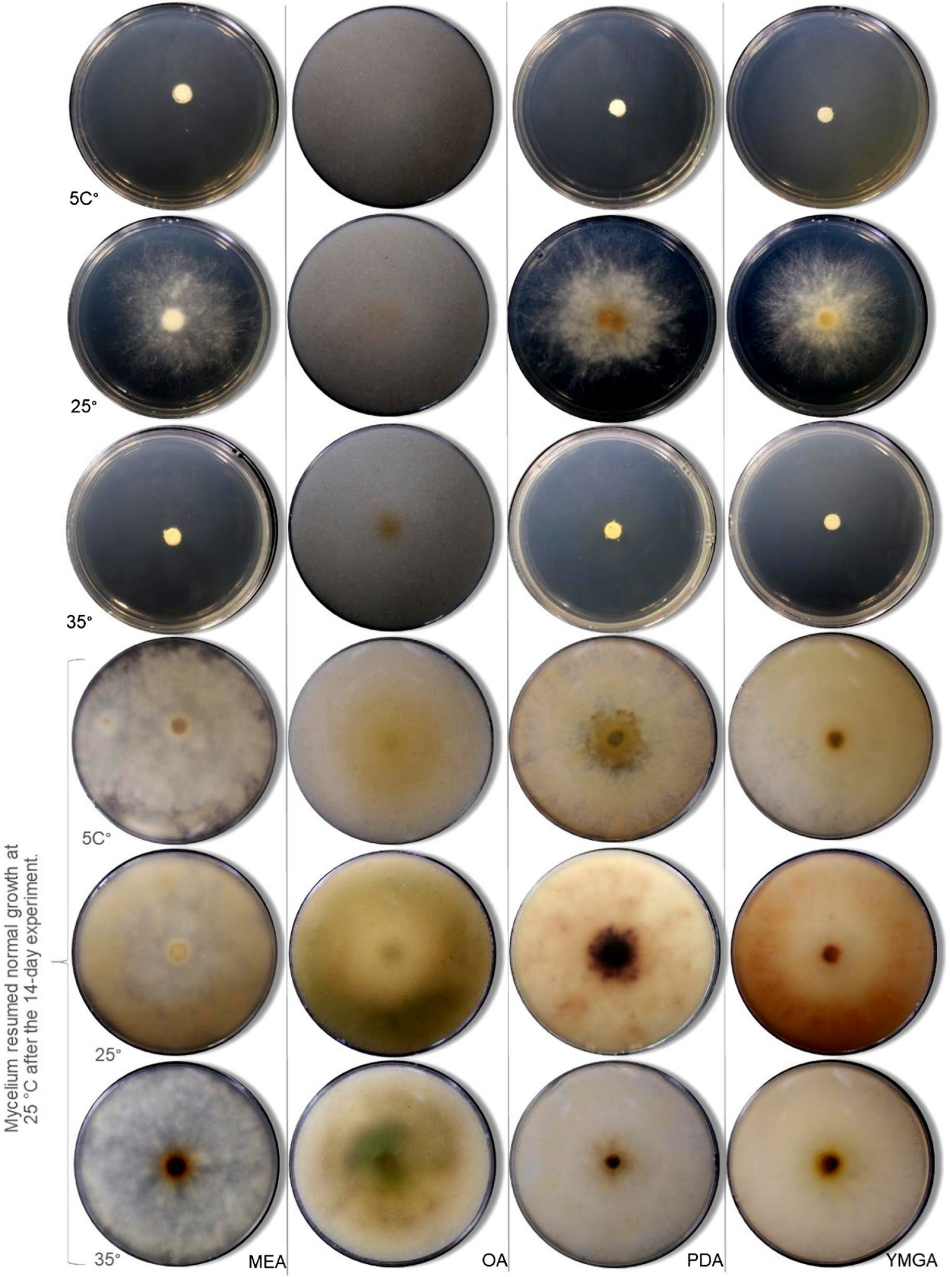
Temperature growth profile of the newly described species after 6 d of incubation on MEA, OA, PDA and YMGA (reverse) at 5 °C, 25 °C, and 35 °C. Normal mycelial growth resumed at 25 °C after incubation under different temperatures 14 days

**Cardinal temperatures**: *Biscogniauxia papillata* grows at 10 °C but not at 5 °C, with optimum growth at 25 °C. Growth is restricted at 35 °C (Figs. 4, 5). After incubating cultures at each test temperature for 14 days, those subsequently returned to the optimum temperature resumed normal growth in all cases, indicating that the species can survive suboptimal thermal conditions and rapidly recover when favorable temperatures are restored. Such thermal resilience suggests that *B. papillata* may be well adapted to withstand short-term temperature extremes predicted under climate change scenarios, enabling persistence in host tissues during unfavorable periods and rapid colonization when conditions improve, a strategy also reported for other members of the genus (Granata and Sidoti 2004).

**Secondary metabolites**: Cyclobiscognioxin A (**1**), and 3,5-dimethyl-8-methoxy-3,4-dihydroisocoumarin (**2**) (Fig. 7).

**Other material examined**: Thailand: Phitsanulok Province, Ban Phaothai Community Forest, 16.733° N, 100.658° E, hill evergreen forest on decaying wood, 22 November 2016, P.S. and S.W. (BCC83050).

### Notes

Morphologically, *Biscogniauxia papillata* resembles *B. communapertura* Y.M. Ju and J.D. Rogers in producing numerous perithecia arranged in rosettes (Fig. 2a and e) and discharging through a single ostiolar canal, with both species also exhibiting papillate ostioles (Fig. 2b–c). In *B. papillata*, two perithecia frequently share a common ostiole (Fig. 2a–b). However, the new species differs from *B. communapertura* in ascospore morphology, being brown to dark brown and significantly larger [(12–)13–15(–16) × (7–)8–9 µm vs. 11.5 × 4.5–6 µm in *B. communapertura*] (Table 2).

Our fungus also resembles *B. plana* (Petch) Y.M. Ju and J.D. Rogers in having numerous perithecia arranged in rosettes and discharging through a single ostiolar canal, but differs by its larger, flask-shaped perithecia [(0.76–)0.90–0.97(–1.01) × (0.18–)0.22–0.30(–0.44) mm vs. 0.50 × 0.90–0.97 mm in *B. plana*] and larger ascospores [(12–)13–15(–16) × (7–)8–9 µm vs. 11–14 × 5.5–7.5 µm].

*Biscogniauxia papillata* shares several features with *B. capnodes* var. *limonispora* Y.M. Ju and J.D. Rogers from Thailand (Ju et al. 1998), including the general stromatal habit. It differs, however, in having papillate ostioles on the stromatal surface, larger ascospores [dark brown, ellipsoid to slightly inequilateral, (12–)13–15(–16) × (7–)8–9 µm vs. 11.5–14 × 6–7.5 µm], and multiple perithecia discharging through a common ostiolar canal. The anamorph also differs: *B. papillata* exhibits a periconiella-like to nodulisporium-like branching pattern in its conidiogenous structures, with the nodulisporium-like form occurring much more frequently, whereas *B. capnodes* var. *limonispora* has a strictly periconiella-like anamorph. Specimens of *B. capnodes* var. *limonispora* from Martinique (Fournier et al. 2017) resemble our collections in gross morphology but differ in ostiolar structure, with *B. papillata* lacking the white ring surrounding the ostioles.

**Fig. 6:**
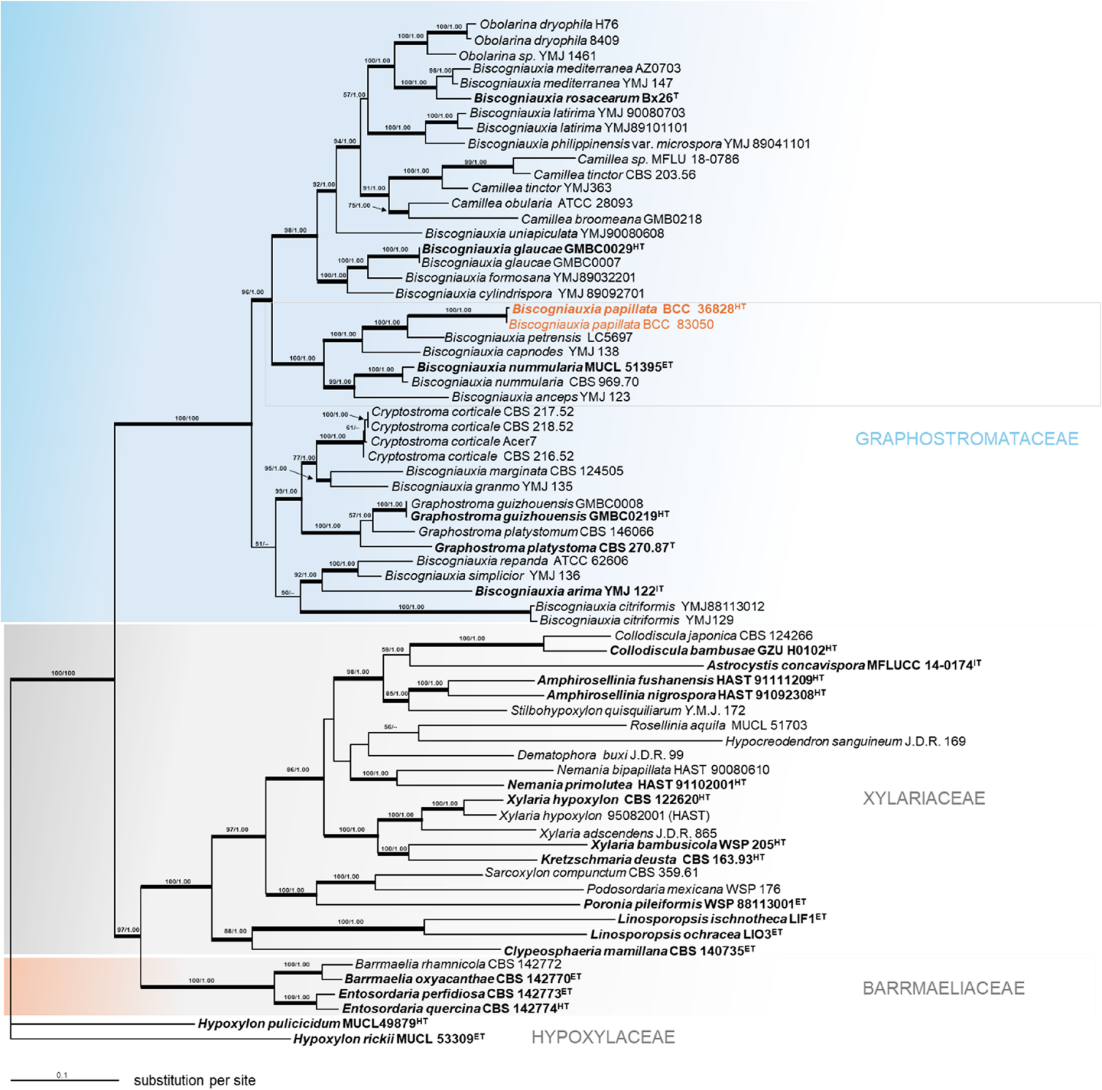
Phylogenetic relationships inferred from RAxML on multi-locus alignment of *Biscogniauxia papillata* and other selected *Xylariales* based on concatenated ribosomal (ITS and LSU) and proteinogenic (*TUB2* and *RPB2*) DNA sequence data. Support values from ML and MB analyses higher than 50% ML and 70% MB are shown above the respective branches. Branches of significant support bootstrap (BS) 70% and posterior probability (PP) 95% are thickened. The sequences of *B. papillata* are highlighted in orange font. ET (ex-epitype), IT (ex-isotype), HT (ex-holotype), PT (ex-paratype), and T (ex-type) strains are highlighted in bold letters

*Biscogniauxia petrensis* Z.F. Zhang, F. Liu and L. Cai produces conidia of similar length [4.5–7.5 × 2.5–4.5 µm vs. (4–)5–6(–7) × (2–)3–4 µm in *B. papillata*], but multi-locus phylogenetic analyses clearly separate the two species with high support (100/1.00), despite their genetic proximity (Fig. 6). Multi-locus phylogenetic analyses also clearly distinguish *B. papillata*, *B. capnodes* (Berk.) Y.M. Ju & J.D. Rogers, *B. nummularia* (Bull.) Kuntze, and *B. anceps* (Sacc.) J.D. Rogers, Y.M. Ju and Cand. into distinct, well-supported lineages.

In terms of morphology, *B. papillata* differs from *B. capnodes* in having larger, flask-shaped perithecia [(0.76–)0.90–0.97(–1.01) × (0.18–)0.22–0.30(–0.44) mm vs. 0.4–0.8 × 0.2–0.4 mm], papillate ostioles lacking the white rim sometimes present in *B. capnodes*, and larger asci [(86–)105–123 µm vs. 70–115 µm] with a more robust apical apparatus [4–5 × 1–2 µm vs. 1.5–4 × 3.5–4 µm]. Its ascospores are broadly ellipsoid, often inequilateral, and larger [(12–)13–15(–16) × (7–)8–9 µm vs. 8.5–15 × 5–7.5 µm], and the anamorph is predominantly nodulisporium-like, with the periconiella-like branching patterns encountered less frequently. The frequent occurrence of two perithecia sharing a common ostiolar canal, absent in *B. capnodes*, is a consistent diagnostic feature.

*Biscogniauxia papillata* is also readily distinguished from *B. nummularia* by stromatal morphology and ascospore form. *Biscogniauxia nummularia* produces applanate stromata with woody, greyish brown to dark brown tissue between the perithecia, whereas *B. papillata* has distinctly bipartite, effused-pulvinate stromata with softer-textured grey tissue beneath the perithecia and a blackish line demarcating the stromatal base from the substrate. The ascospores of *B. nummularia* are smaller, nearly equilateral, and more rounded [10–13(–14) × (6.5–)7.5–8.5 µm], contrasting with the larger, broadly ellipsoid and often inequilateral ascospores of *B. papillata*. Biogeographically, *B. nummularia* is largely restricted to Europe, *B. capnodes* is rarely encountered there (Ju et al. 1998), and *B. papillata* is presently known only from Thailand.

*Biscogniauxia anceps* is clearly distinct from *B. papillata* both molecularly and morphologically. It forms applanate, discoid to widespreading stromata with distinct margins and a cracked surface reminiscent of *Diatrype stigma* (Ju et al. 1998), smaller perithecia (0.2–0.4 mm diam), and umbilicate ostioles within grey depressed areas. Its ascospores are unequally two-celled, with the larger cell sometimes dark brown but most commonly hyaline, and the smaller cell always hyaline without a germ slit; in contrast, *B. papillata* produces uniseptate, dark brown, broadly ellipsoid ascospores with a straight, full-length germ slit, larger flask-shaped perithecia, and an anamorph that is most often nodulisporium-like, with the periconiella-like branching patterns occurring less frequently.

### Molecular phylogenetic inference

After providing full taxonomic description of the newly introduced fungus from Thailand, we confirmed its phylogenetic placement using multi-locus DNA analyses as shown in Fig. 6. The six newly generated ITS, LSU, and *RPB2* sequences were compared with data from GenBank NCBI nucleotide database (PCR amplifications yielded approximately 500 bp, 1000 bp, 800 bp, and 1000 bp of ITS rDNA, LSU rDNA, *RPB2*, and *TUB2* sequences, respectively). The phylogenetic relationships were estimated using the ML and MB analyses. The dataset of the multi-locus DNA sequences included 69 taxa from *Amphirosellinia* (2), *Astrocystis* (1), *Barrmaelia* (2), *Biscogniauxia* (27), *Camillea* (5), *Dematophora* (1), *Clypeosphaeria* (1), *Collodiscula* (2), *Cryptostroma* (4), *Entosordaria* (2), *Graphostroma* (4), *Hypocreodendron* (1), *Hypoxylon* (2), *Kretzschmaria* (1), *Linosporopsis* (2), *Nemania* (2), *Obolarina* (3), *Podosordaria* (2), *Rosellinia* (1), *Sacroxylon* (1), *Stilbohypoxylon* (1), and *Xylaria* (4). The best phylogenetic tree inferred from RAxML had a likelihood of − 71969.270958. The alignment had 2969 distinct alignment patterns, with 39.98% undetermined characters or gaps. Estimated base frequencies were as follows: 0.237200, *C* = 0.273463, *G* = 0.246760, *T* = 0.242577; substitution rates were AC = 1.265867, AG = 3.551300, AT = 1.172200, CG = 0.892001, CT = 5.053569, GT = 1.000; gamma distribution shape parameter was α 0.340307. The likelihood of the Bayesian tree was − 82154.19. As shown in Fig. 6, the sequences of our new species are well separated from the previously reported species in *Biscogniauxia* that have been recently updated by Li et al. (2021). The topology of the RAxML tree is practically identical to the one presented by Wendt et al. (2018) and Samarakoon et al. (2022).

### Secondary metabolite profiling and isolation

After analyzing the secondary metabolite production of *B. papillata*, we decided to scale up its cultivation in BRFT solid medium. Among the major components, a metabolite of medium polarity with high molecular weight was initially assumed to be related to sansalvamide A, a compound first reported from a marine *Fusarium* sp., with a similar derivative previously identified in *B. mediterranea* (Belofsky et al. 1999; Wu et al. 2016). To confirm the identity of this compound, we targeted its isolation. Consequently, compound **1** was purified as an amorphous solid, whose HR-ESI–MS spectrum established its molecular formula as C_33_H_53_N_5_O_5_ by revealing a protonated molecular ion peak at *m/z* 600.4118 [M + H]^+^ (calculated 600.4125 for C_33_H_54_N_5_O_5_^+^) indicating ten degrees of unsaturation. The ^13^C NMR spectral data of **1** (Table S1, Figure S4) revealed the presence of five carbonyl carbon atoms at δ_C_ 174.64, 174.58, 174.1, 174.0, and 173.9 ppm that suggested, together with its ^1^H NMR spectral data (Table S1, Figure S3), being a cyclopentapeptide derivative. The ^1^H NMR spectral data of **1** (Table S1, Figure S3) revealed five α proton signals at δ_H_ 3.29 ~ 4.67 ppm; four *β*-diastereotopic methylene groups at δ_H_ 1.42/1.49, 1.57/1.64, 1.56/1.70, and 2.88/3.02 ppm and one *β*-methine proton at δ_H_ 2.38 ppm. In addition, the ^1^H NMR spectral data of **1** also revealed five aromatic protons (δ_H_ 7.20 ~ 7.27 ppm) denoting a monosubstituted aromatic ring together with three pairs of doublet methyl groups at δ_H_ 0.99/0.95, 0.97/0.89, and 0.90/0.85; one triplet methyl at δ_H_ 0.87; and a doublet methyl group at δ_H_ 0.72 ppm. Based on the obtained results, compound **1** was suggested to be a cyclic pentapeptide consisting of one phenylalanine, three leucine and one isoleucine residues. A literature search of **1** revealed its close similarity to a previously reported cyclopentapeptide that was isolated from the methanol extract of liquid culture medium mycelia of an unidentified endophytic fungal strain no. 2524 derived from a seed of *Avicennia marina*, a mangrove plant in Hongkong (Li et al. 2004). A careful comparison of ^13^C/^1^H NMR data of both **1** and *cyclo*-(L-Phe-L-Leu^1^-L-Leu^2^-L-Leu^3^-L-Ile) (Li et al. 2004) (Table S1) revealed that they are virtually identical. The amino acid sequence of **1** was established via acquiring its HMBC spectrum that revealed key correlations from αH of Phe at δ_H_ 4.67 to the carbonyl group of Leu^1^ at δ_C_ 174.0 and αH signals of Leu^1^-Leu^3^ at δ_H_ 4.44, 4.27, and 4.21 to carbonyl groups of Leu^2^, Leu^3^, and Ile at 174.58, 174.64, and 174.1 ppm, respectively. The αH of Ile at δ_H_ 3.29 revealed key HMBC correlation to the carbonyl group of Phe at δ_C_ 173.9 thus confirming its presence as a cyclic pentapeptide of Phe-Leu^1^-Leu^2^-Leu^3^-Ile. The absolute configuration of amino acid residues was determined via Marfey’s method as previously described (Wennrich et al. 2024; Holzenkamp et al. 2024). The results of Marfey’s method (data not shown) determined that all amino acids in **1** are present in L configurations. Thus, compound **1** was unambiguously characterized as *cyclo*-(L-Phe-L-Leu^1^-L-Leu^2^-L-Leu^3^-L-Ile) and it was given a trivial name cyclobiscognioxin A.

**Fig. 7:**
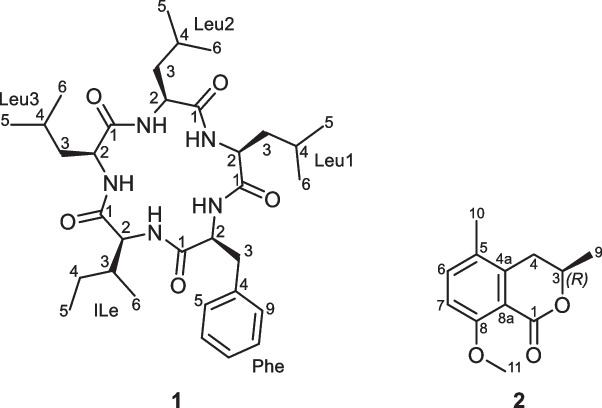
Chemical structures of secondary metabolites isolated from the solid cultures of *Bicogniauxia papillata*, cyclobiscognioxin A (**1**), and (3*R*)−3,5-dimethyl-8-methoxy-3,4-dihydroisocoumarin (**2**)

Compound **2** was obtained as a colorless amorphous solid. The HR-ESI–MS spectrum of **2** revealed a protonated molecular ion and a sodium adduct at *m/z* 207.1017 (calculated 207.1021) and 229.0837 (calculated 229.0841), respectively. Thus, the molecular formula of **2** was determined as C_12_H_14_O_3_ indicating six degrees of unsaturation. The ^1^H NMR spectral data of **2** (Table S2, Figure S11) revealed two *ortho*-coupled aromatic proton signals at δ_H_ 7.40 and 6.96 ppm with a coupling constant (*J* value) of 8.6 Hz; an aliphatic methine at δ_H_ 4.46 (dqd, *J* = 11.4, 6.4, 2.8 Hz); a diastereotopic methylene group at δ_H_ 2.63/2.95 and three methyl groups including one doublet at δ_H_ 1.38 (d, *J* = 6.4 Hz), two singlets at δ_H_ 2.19 and 3.78 ppm. A literature search of **2** revealed its close resemblance to 3,5-dimethyl-8-methoxy-3,4-dihydroisocoumarin (aka 5-methylmellein) that was previously reported from a culture filtrate of *Cytospora eucalypticola* (Kokubun et al. 2003). To further confirm the depicted structure of **2** (Fig. 7), its 2D NMR spectra as ^1^H-^1^H COSY, HMBC, and HSQC (Figures S12–S15) were recorded. The ^1^H-^1^H COSY spectrum (Figures S12 and S15) revealed two spin systems: one between two *ortho*-coupled aromatic protons (H-6/H-7) and another spin system extends a diastereotopic methylene group (H_2_−4) to a methine proton (H-3) and ends at a double methyl moiety (H_3_−9). The HMBC spectrum of **2** (Figures S13 and S15) revealed key correlations from H-6 to C-5 (δ_C_ 140.3) and C-8 (δ_C_ 158.6); from an olefinic methyl group H_3_−10 to C-4a (δ_C_ 125.9), C-5 and C-6 (δ_C_ 135.3) confirming its position at C-5. In addition, a singlet methoxy group (H_3_−11) and H-6 to C-8 (δ_C_ 158.6) and from a doublet methyl group (H_3_−9) to a methylene carbon at δ_C_ 32.1 (C-4) confirming their positions at C-8 and C-3 (δ_C_ 72.8), respectively. The absolute configuration at C-3 was determined based on analyzing the coupling constant (*J* value) of H-3 that indicated its existence in axial orientation while the doublet methyl group H_3_-9 to be on equatorial orientation. Hence, the absolute configuration at C-3 was assigned as (3*R*) configuration.

Overall, cyclobiscognioxin A (**1**) was isolated together with the previously reported mellein derivative, (3*R*)−3,5-dimethyl-8-methoxy-3,4-dihydroisocoumarin (5-methylmellein, **2**) (Figure 7). Given the fact that several cyclic peptides are known for their toxicity and diverse ecological roles, we decided to evaluate the antimicrobial and cytotoxic properties of **1**. Our assays identified cyclobiscognioxin A (**1**) as a potent cytotoxic agent but no antimicrobial activity against the tested microorganisms was observed (Table S3). The obtained results revealed that **1** featured the strongest cytotoxic effects against A549 (lung), KB3.1 (cervix), and MCF-7 (breast) cancer cell lines at IC_50_ values of 4.7, 5.2, and 5.5 µM, respectively.

## Discussion

The genus *Biscogniauxia* exhibits diverse ecological roles, including saprobic, endophytic, and pathogenic behaviors on weakened or stressed hosts, as illustrated by reports of disease in oak, almond, and strawberry by some of these taxa (Yangui et al. 2021). Species in this genus are frequently associated with hardwoods, colonizing bark, wood, and decayed plant material (Ju et al. 1998). For example, *B. anceps* occurs on *Quercus* and *Corylus* in temperate regions, whereas tropical congeners, including the species described here, thrive in the tropical forest ecosystems (Ju et al. 1998). Related taxa can also become pathogenic under suitable conditions such as *Cryptostroma corticale*, a close relative within the *Graphostromataceae*, which causes the sooty bark disease on sycamore (*Acer pseudoplatanus*) (Brenken et al. 2024). Motivated by these considerations, we evaluated whether *B. papillata* can grow outside its optimal temperature range, recognizing that climate warming and derived drought are likely to alter distributions, fitness, and host interactions by stressing trees and ultimately increasing susceptibility to opportunistic infections (Nugent et al. 2005; Desprez-Loustau et al. 2006). During our experiments, mycelial growth was optimal at 25 °C with intact hyphal structures, but growth declined sharply at ≤ 10 °C, consistent with persistence despite reduced competitive capacity (Lan et al. 2025). Growth was also slowed above 30 °C, which coincided with the development of the pigmentation on agar media (Fig. 4), suggesting a stress-mitigation response (Venkatachalam et al. 2019). Similar adaptability has been reported in *B. mediterranea*, which can grow from 5 to 45 °C, with an optimum near 35 °C, and recovers rapidly when conditions improve (Henriques et al. 2015; Bakhshi-Ganje et al. 2024). This pattern indicates a broader capacity across *Biscogniauxia* species to exploit variable thermal regimes. Altogether, these observations support a bet-hedging strategy in which prolonged, low-activity endophytism shifted to opportunistic pathogenicity during occasional heatwaves and drought.

To place these findings in a taxonomic context, we constructed a multi-locus phylogeny, which was consistent with Ju et al. (1998) morphological studies and with recent multi-locus analyses that delimited the species in the family *Graphostromataceae* and its constituent genera, *Biscogniauxia*, *Camillea*, *Cryptostroma*, *Graphostroma*, *Obolarina*, and *Vivantia* (Wendt et al. 2018; Daranagama et al. 2018). In the combined-sequence analyses, our new collections form a well-supported clade within the lineage that includes *B. capnodes* and closely related taxa, reinforcing their placement in *Biscogniauxia* sensu lato. Our phylogenetic analyses further confirmed the distinction between *B. capnodes* and *B. nummularia* and were consistent with Ju et al.’s (1998) morphological studies that differentiated these species based on stromatal characteristics and ascospore morphology. *Biscogniauxia capnodes* is characterized by carbonaceous tissue between the perithecia and ellipsoid ascospores, whereas *B. nummularia* possesses grey to brown woody tissue between the perithecia and more rounded ascospores Ju et al. (1998). Geographically, *B. nummularia* is primarily found in Europe, while *B. capnodes* is rarely recorded in the region (Ju et al. 1998). Similarly, *B. anceps* is phylogenetically distinct from our new species, consistent with their morphological concept. Both differ in their ascospore structure and their ecology. While *B. papillata* has coalescent stromata, flattened perithecia with shared ostioles, and larger, lemon-shaped ascospores, *B. anceps* has smaller, two-celled ascospores, with the larger cell measuring 10–13 µm and the smaller cell 5–7 µm, typically ellipsoid to obovate in shape. In addition, *B. anceps* is primarily distributed in temperate regions, including Italy and France (Ju et al. 1998). *Biscogniauxia papillata* is closely related to *B. capnodes*, which agrees with their well-defined morphological features. However, our new species is distinguished by its smaller, ellipsoid-lobed, coalescent stromata with a dehiscing layer, carbonaceous tissue beneath the perithecia, and flattened perithecia with some sharing a common ostiole, characteristics rarely found in *B. capnodes*. Additionally, *B. papillata* exhibits larger, lemon-shaped ascospores with slightly pinched ends and a spore-length germ slit, giving this fungus a unique identity among its relatives. According to the phylogenetic analyses, *B. papillata* is clearly separated from *B. petrensis.* The latter was first reported in its anamorphic state and shown to form a sister group with *B. capnodes*, as the species was isolated from rock in a karst cave in China (Zhang et al. 2017). While the conidial features of our new species resemble those of *B. petrensis*, samples isolated from the roots of *Dendrobium harveyanum* in Thailand (Ma et al. 2020) were morphologically similar to those from karst caves in China. However, their distinction is supported by our molecular phylogeny, which provides strong support from both RAxML and MrBayes analyses.

Molecular phylogenetic analyses clearly separate our new species from *Camillea*, which, as defined by Læssøe et al. (1989) and further studied by Fournier et al. (2023), is characterized by highly carbonaceous stromata erumpent through bark with a fleeting ectostromatic layer of mixed bark and fungal tissues, asci bearing a massive rhomboid amyloid apical apparatus, subhyaline to yellowish ornamented ascospores visible under SEM, and a xylocladium-like anamorph. In contrast, *B. papillata* develops bipartite, effused-pulvinate stromata without an ectostromatic layer, asci with a smaller discoid apical apparatus, dark brown smooth-walled ascospores, and an anamorph that is predominantly nodulisporium-like with a less common periconiella-like form, and is consistently resolved within *Biscogniauxia* with strong statistical support.

The isolation of compound **1** as the major secondary metabolite of *B. papillata* is particularly relevant for the study of related species, especially given its observed *in vitro* toxicity in our assays. Cyclobiscognioxin A (**1**) showed potent, dose-dependent cytotoxicity with no detectable antimicrobial activity against the test pane l. This bioactivity profile aligns with hydrophobic cyclic pentapeptides that act in eukaryotic cells, often transiting membranes as “molecular chameleons” and defying small-molecule rule-sets (Buckton et al. 2021). Given the latent pathogenicity observed in some *Biscogniauxia* species and their parasitic status, the production of toxic metabolites might represent adaptive mechanisms associated to host interactions, competition, or environmental stress responses (Patejuk et al. 2022; Nugent et al. 2005). Cyclic peptides often exhibit bioactive properties, including cytotoxic, antifungal, and antibacterial effects (Ribeiro et al. 2022), suggesting roles in shaping fungal community dynamics and host–pathogen interactions. Destruxins, produced by *Metarhizium* species, contribute to fungal virulence against insect hosts by suppressing immune responses (Kobmoo et al. 2024), while beauvericins from *Fusarium* exhibit cytotoxic and antimicrobial activities that might influence fungal competition and pathogenicity (Logrieco et al. 1998). We recently showed in *Cordyceps* spp. that diverse cyclic peptides, including beauveriolides and beauvericins, accumulate in insect cadavers, supporting a role during infection (Charria-Girón et al.^2023^). Future chemotaxonomic studies within *Graphostromataceae* are essential to uncover the cryptic chemistry of this fungal family. A detailed evaluation of the biological properties of cyclobiscognioxin A (**1**) and related compounds is required to clarify their ecological roles and potential as a pathogenicity factor.

Recent phylogenomic studies have provided deeper insights into the evolutionary dynamics of *Biscogniauxia*, revealing extensive gene duplications and horizontal gene transfers (HGTs), particularly in relation to secondary metabolite gene clusters (SMGCs) (Franco et al. 2022). These genetic adaptations may have played a crucial role in the ecological versatility of *Biscogniauxia*, allowing species within the genus to act as both saprotrophs and opportunistic pathogens, depending on environmental conditions. Such evolutionary mechanisms highlight the significance of *Biscogniauxia* in forests, where it participates in both wood decay processes and plant-pathogen interactions (Ju et al. 1998; Henriques et al. 2014; 2016). This complexity is reflected in the ecological versatility of the genus, with species capable of shifting between saprotrophic and opportunistic pathogenic lifestyles depending on environmental conditions. Such adaptive strategies are particularly relevant in the context of fungal systematics, as they offer insight into the ecological roles and evolutionary history of *Biscogniauxia*. Concurrently, we are expanding sampling across Thailand and Southeast Asia to build a population-level framework for precise species delimitation, combining genotyping and multi-locus phylogeny. These data will be integrated with state-of-the-art metabolomics to delineate chemotaxonomic traits and map them onto the evolutionary history of the *Xylariales*. We also plan controlled host–interaction assays to examine the endophyte-to-pathogen transition and identify candidate virulence factors. Finally, we will establish an open reference library for Thai *Biscogniauxia,* including vouchers, living cultures, and curated metabolite profiles, to enable reproducible identification and support responsible bioprospecting by the broader mycological community.

## Supplementary Information

Below is the link to the electronic supplementary material.

ESM 1 (PDF 2.27 MB)

## Data Availability

All sequence data generated for this work can be accessed via GenBank: https://www.ncbi.nlm.nih.gov/genbank/.
