# Supplementary material for: Polyphasic characterization of Biscogniauxia papillata sp. nov. (Graphostromataceae) and isolation of the cytotoxic cyclic pentapeptide cyclobiscognioxin A
Source: Mycol Prog. 2026 Jan 8;25(1):2. doi: 10.1007/s11557-025-02114-y (PMC12783183; doi:10.1007/s11557-025-02114-y)
Supplement: Supplementary file 1 — (PDF 2.27 MB) [file 11557_2025_2114_MOESM1_ESM.pdf]

Supporting Information for:

Polyphasic characterization of *Biscogniauxia papillata* sp. nov. (*Graphostromataceae*) and isolation of the cytotoxic cyclic pentapeptide cyclobiscognioxin A

Sarunyou Wongkanoun[1,2], Esteban Charria-Girón[3,4]*, Boonchuai Chainuwong[5], Prasert Srikitikulchai[5], Natapol Pornputtapong[1], Jennifer Luangsa-ard[2], Sherif S. Ebada[3,6]*, Marc Stadler [3,7]*

[1]Department of Biochemistry and Microbiology, Center of Excellence for DNA Barcoding of Thai Medicinal Plants, Faculty of Pharmaceutical Sciences, Chulalongkorn University, Bangkok 10330, Thailand

[2]Plant Microbe Interaction Research Team (APMT), National Center for Genetic Engineering and Biotechnology (BIOTEC), 113 Thailand Science Park, Phahonyothin Road, Khlong Nueng, Khlong Luang, Pathum Thani 12120, Thailand

[3]Department of Microbial Drugs, Helmholtz Centre for Infection Research GmbH (HZI), German Centre for Infection Research Association (DZIF), partner site Hannover-Braunschweig, Inhoffenstraße 7, 38124 Braunschweig, Germany

[4]Bioinformatics Group, Wageningen University & Research, Droevendaalsesteeg 1, 6708 PB Wageningen, the Netherlands

[5]National Biobank of Thailand (NBT), National Center for Genetic Engineering and Biotechnology (BIOTEC), 111 Thailand Science Park, Phahonyothin Road, Khlong Nueng, Khlong Luang, Pathum Thani 12120, Thailand

[6]Department of Pharmacognosy, Faculty of Pharmacy, Ain Shams University, 11566 Cairo, Egypt

[7]Institute of Microbiology, Technische Universität Braunschweig, Spielmannstraße 7, 38106 Braunschweig, Germany

*Corresponding Author: esteban.charriagiron@helmholtz-hzi.de; esteban.charriagiron@wur.nl (E.C.-G.); sherif.elsayed@helmholtz-hzi.de (S.S.E.); Marc.Stadler@helmholtz-hzi.de (M.S.); Tel.: +49-531-6181-424; Fax +49-531-6181-9499

Table of Content

#	Contents	Page
**1**	Figure S1. LR-ESI-MS of **1**.	**S3**
**2**	Figure S2. HR-ESI-MS of **1**.	**S4**
**3**	Figure S3. ^{1}H NMR spectrum of **1** in methanol-d_4 at 500 MHz.	**S5**
**4**	Figure S4. ^{13}C NMR spectrum of **1** in methanol-d_4 at 125 MHz.	**S6**
**5**	Figure S5. ^{1}H–^{1}H NMR spectrum of **1** in methanol-d_4 at 500 MHz.	**S7**
**6**	Figure S6. HMBC spectrum of **1** in methanol-d_4 at 500 MHz.	**S8**
**7**	Figure S7. HSQC spectrum of **1** in methanol-d_4 at 500 MHz.	**S9**
**8**	Figure S8. ROESY spectrum of **1** in methanol-d_4 at 500 MHz.	**S10**
**9**	Table S1. ^{1}H and ^{13}C NMR data of **1**.	**S11**
**10**	Figure S9. LR-ESI-MS of **2**.	**S12**
**11**	Figure S10. HR-ESI-MS of **2**.	**S13**
**12**	Figure S11. ^{1}H NMR spectrum of **2** in DMSO-d_6 at 500 MHz.	**S14**
**13**	Figure S12. ^{1}H-^{1}H COSY spectrum of **2** in DMSO-d_6 at 500 MHz.	**S15**
**14**	Figure S13. HMBC spectrum of **2** in DMSO-d_6 at 500 MHz.	**S16**
**15**	Figure S14. HSQC spectrum of **2** in DMSO-d_6 at 500 MHz.	**S17**
**16**	Figure S15. Key ^{1}H-^{1}H COSY and HMBC correlations of **2**.	**S18**
**17**	Table S2. ^{1}H and ^{13}C NMR data of **2** and 3,5-dimethyl-8-hydroxy-7-methoxy-3,4-dihydroisocoumarin.	**S18**
**18**	Table S3. Cytotoxicity (IC_{50}) and antimicrobial activity (MIC) of **1** and **2**.	**S19**

Display Report

Analysis Info

Analysis Name	S:\DATA\AmaZon\swo23-Bank Wongkanoun\2024\Esteban\XY01542\XY01542_F10_RICE_Ethyl_Acetate_GC2_01_17779.d	Acquisition Date	17.07.2024 04:03:32
Method	17779.m	Operator	lab
Sample Name	XY01355_F10_RICE_Ethyl_Acetate	Instrument	amaZon speed
Comment			

Acquisition Parameter

Ion Source Type	ESI	Ion Polarity	Negative	Alternating Ion Polarity	on
Mass Range Mode	UltraScan	Scan Begin	100 m/z	Scan End	2000 m/z
Accumulation Time	2339 µs	RF Level	100 %	Trap Drive	68.9
SPS Target Mass	1000 m/z	Averages	6 Spectra		

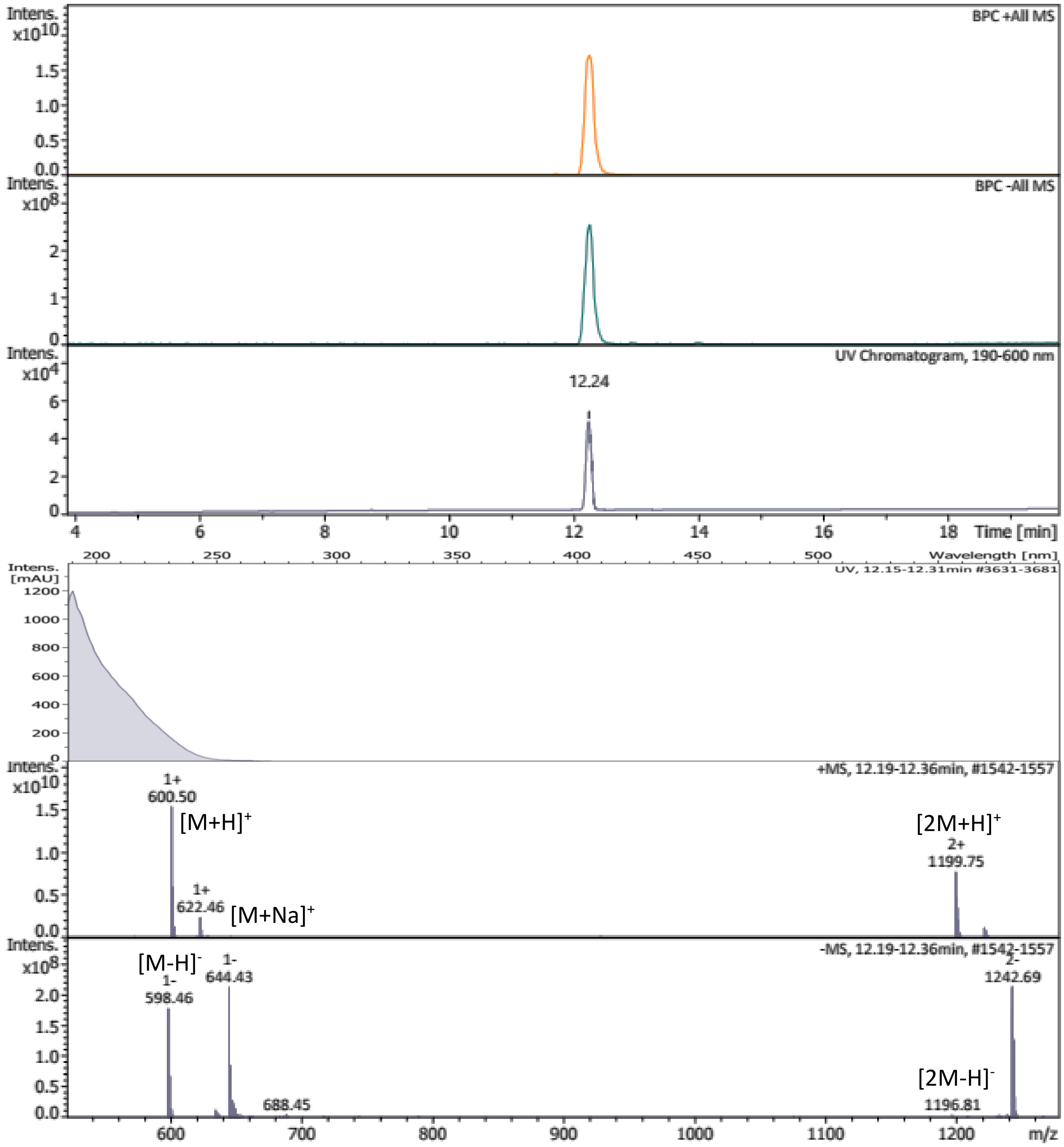

Bruker Compass DataAnalysis 6.1 printed: 12.09.2024 06:49:38 by: sel22 Page 1 of 1

Figure S1. LR-ESI-MS of **1**.

Display Report

Analysis Info

Analysis Name	S:\DATA\timsTOF\SWO24_Bank_SarunyouWongkanoun\24_08\XY01542-F10_P1-B-5_1_4015.d	Acquisition Date	08.08.2024 15:38:54
Method	MWIS-4D-Metabolomics_mz85-1500.m	Operator	Admin
Sample Name	XY01542-F10	Instrument	timsTOF Pro 2
Comment			

Acquisition Parameter

Ion Polarity Positive

SPS Target Mass

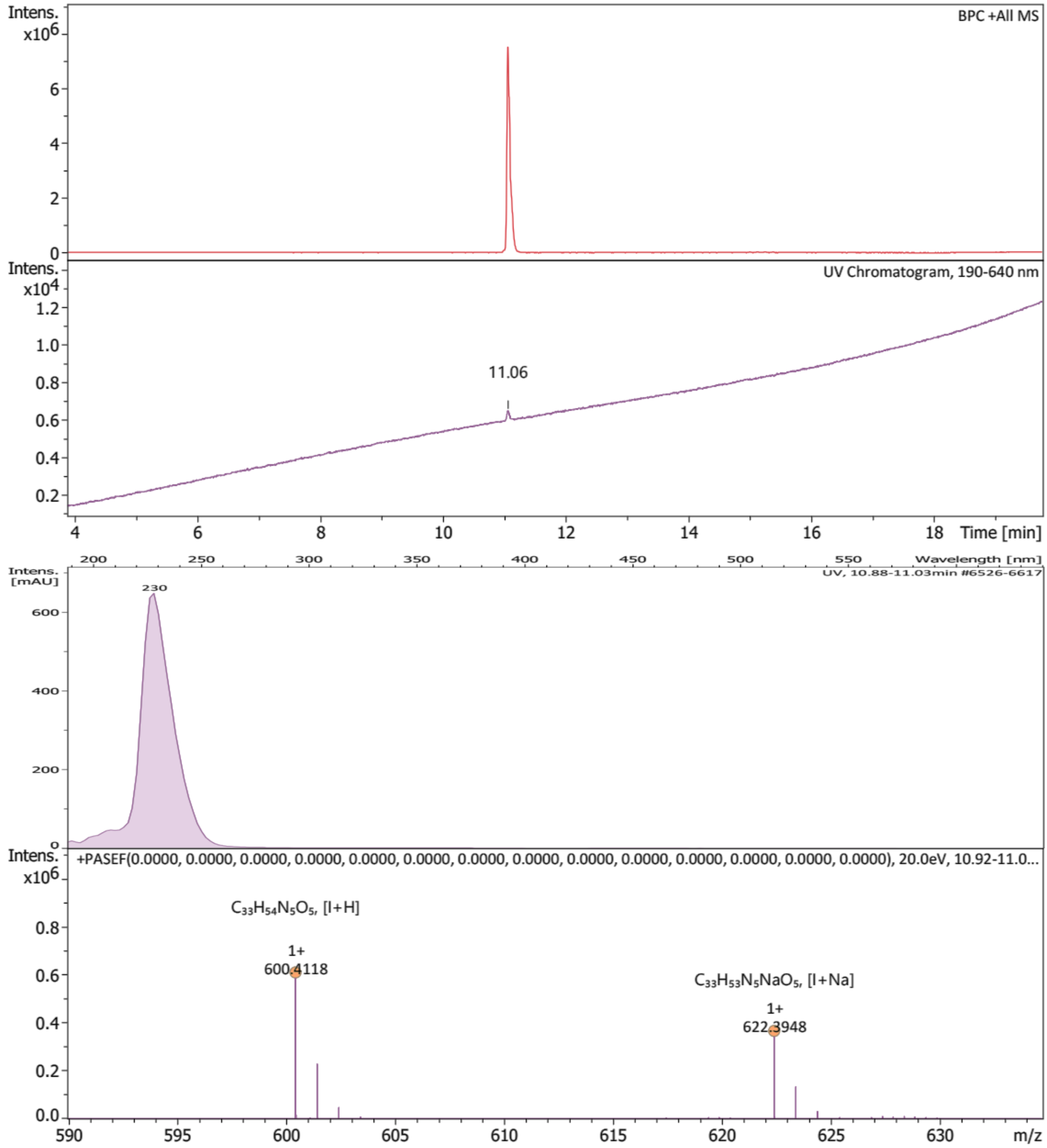

Figure S2. HR-ESI-MS of **1**.

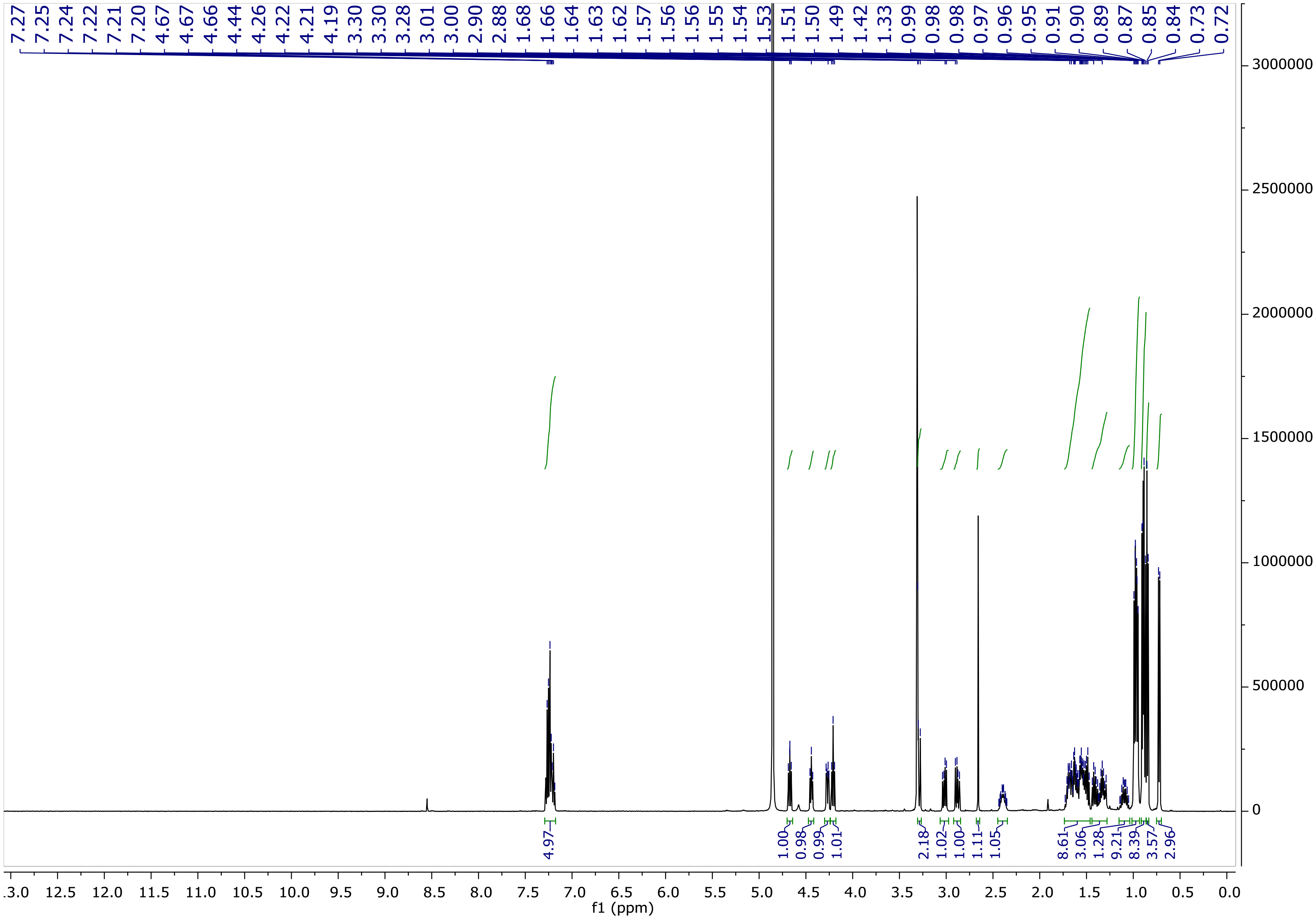

Figure S3. ^{1}H NMR spectrum of **1** in methanol-d_4 at 500 MHz.

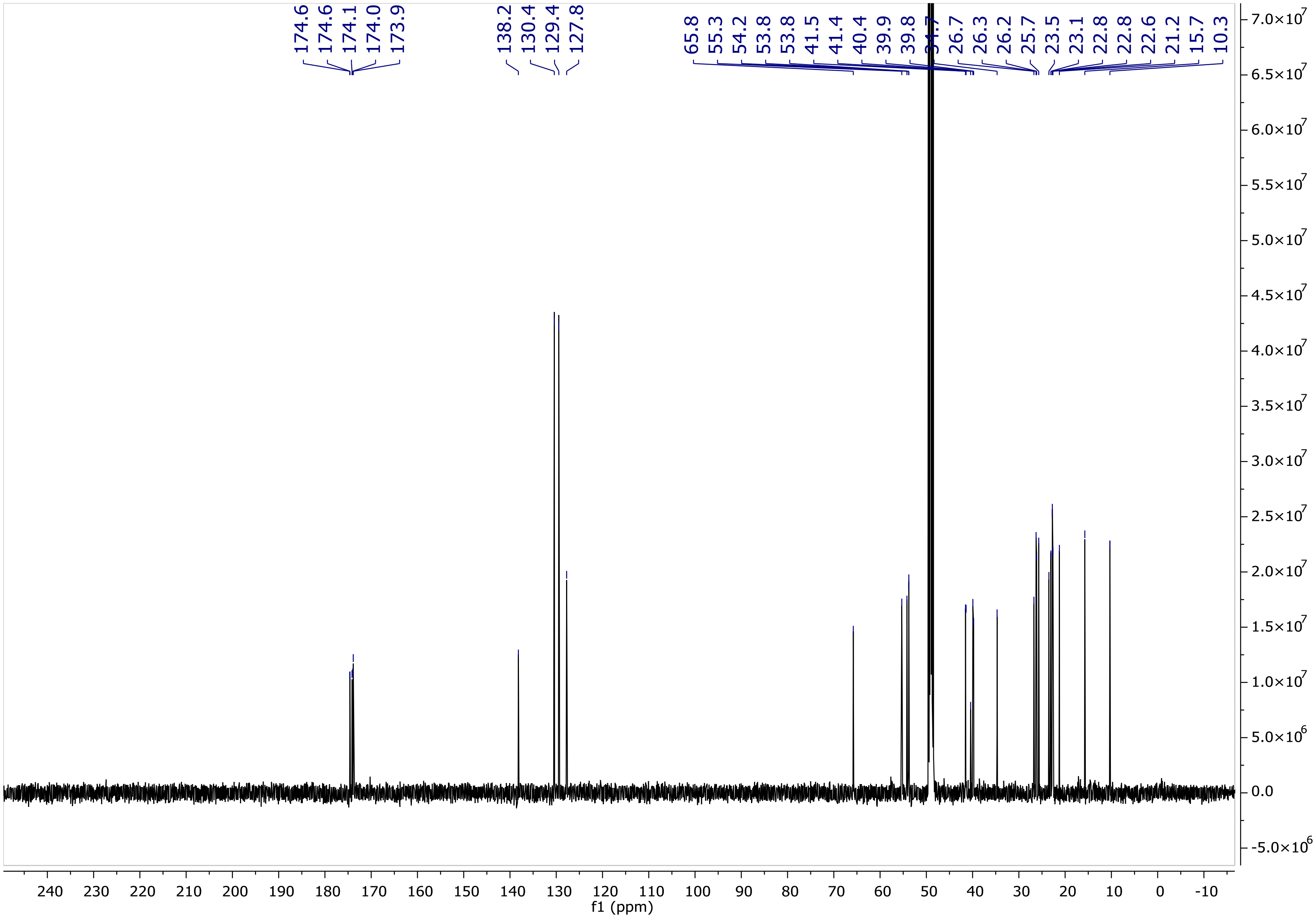

Figure S4. ^{13}C NMR spectrum of **1** in methanol-d_4 at 125 MHz.

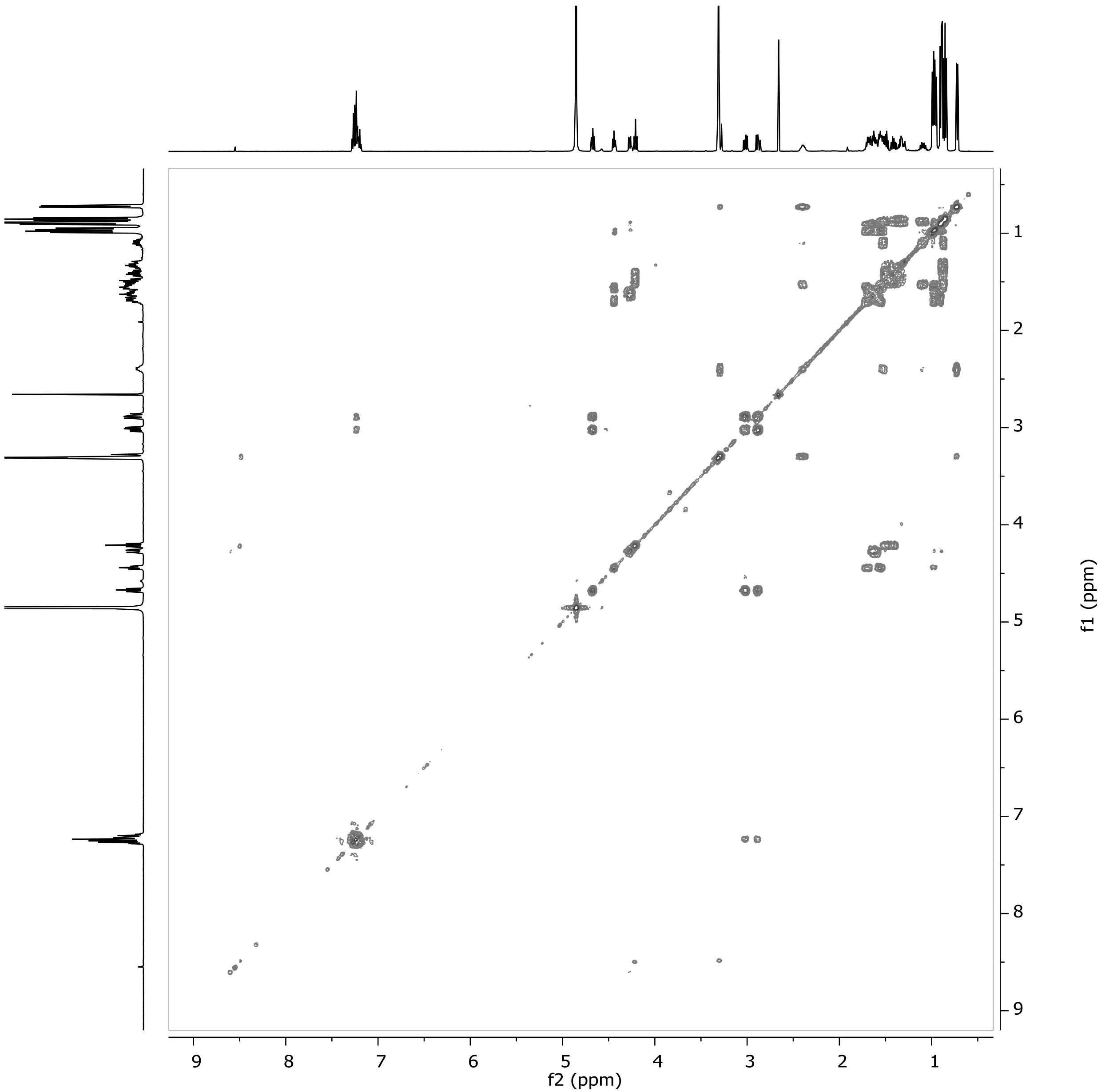

Figure S5. ^{1}H–^{1}H NMR spectrum of **1** in methanol-d_4 at 500 MHz.

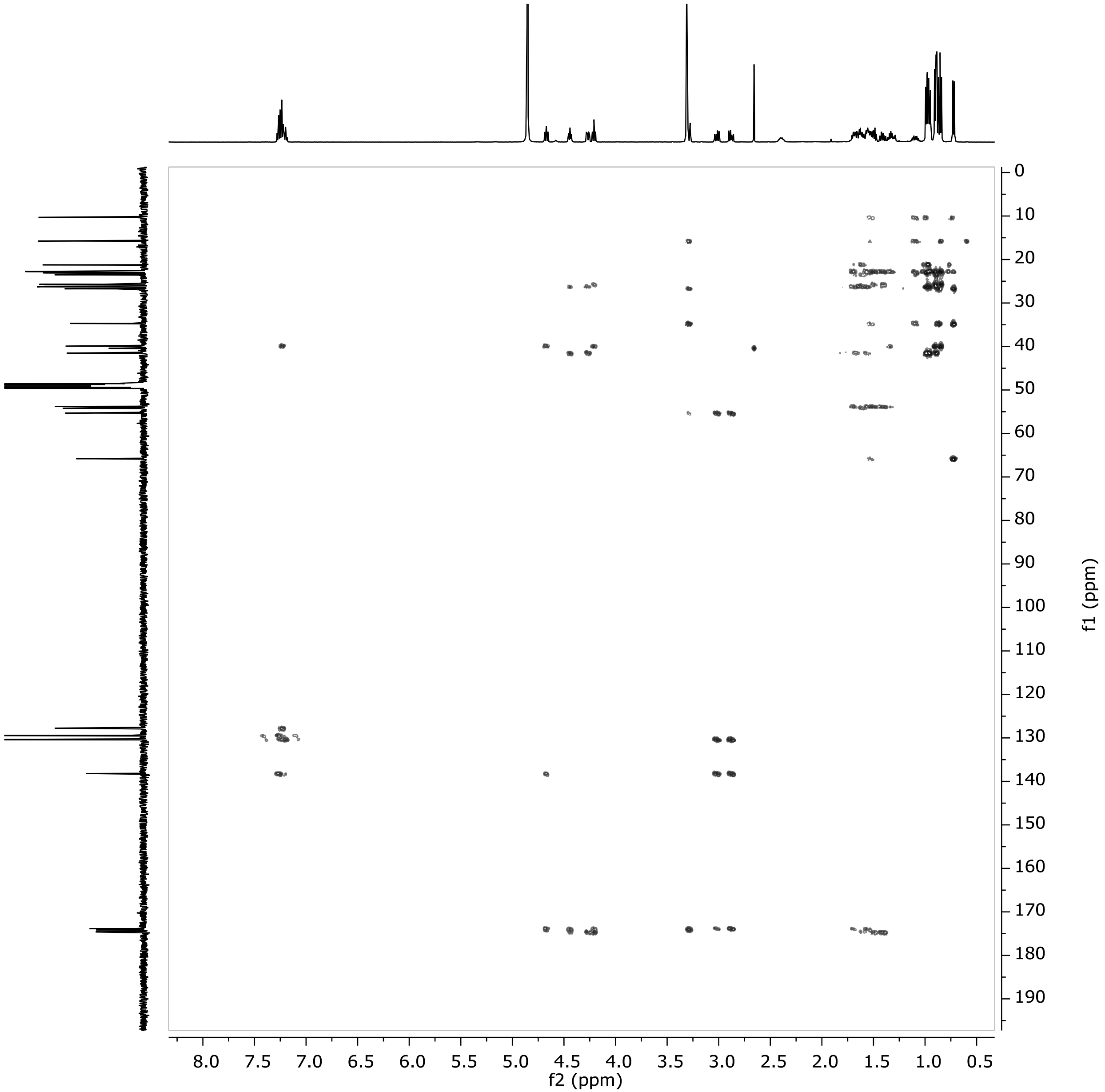

Figure S6. HMBC spectrum of **1** in methanol-d_4 at 500 MHz.

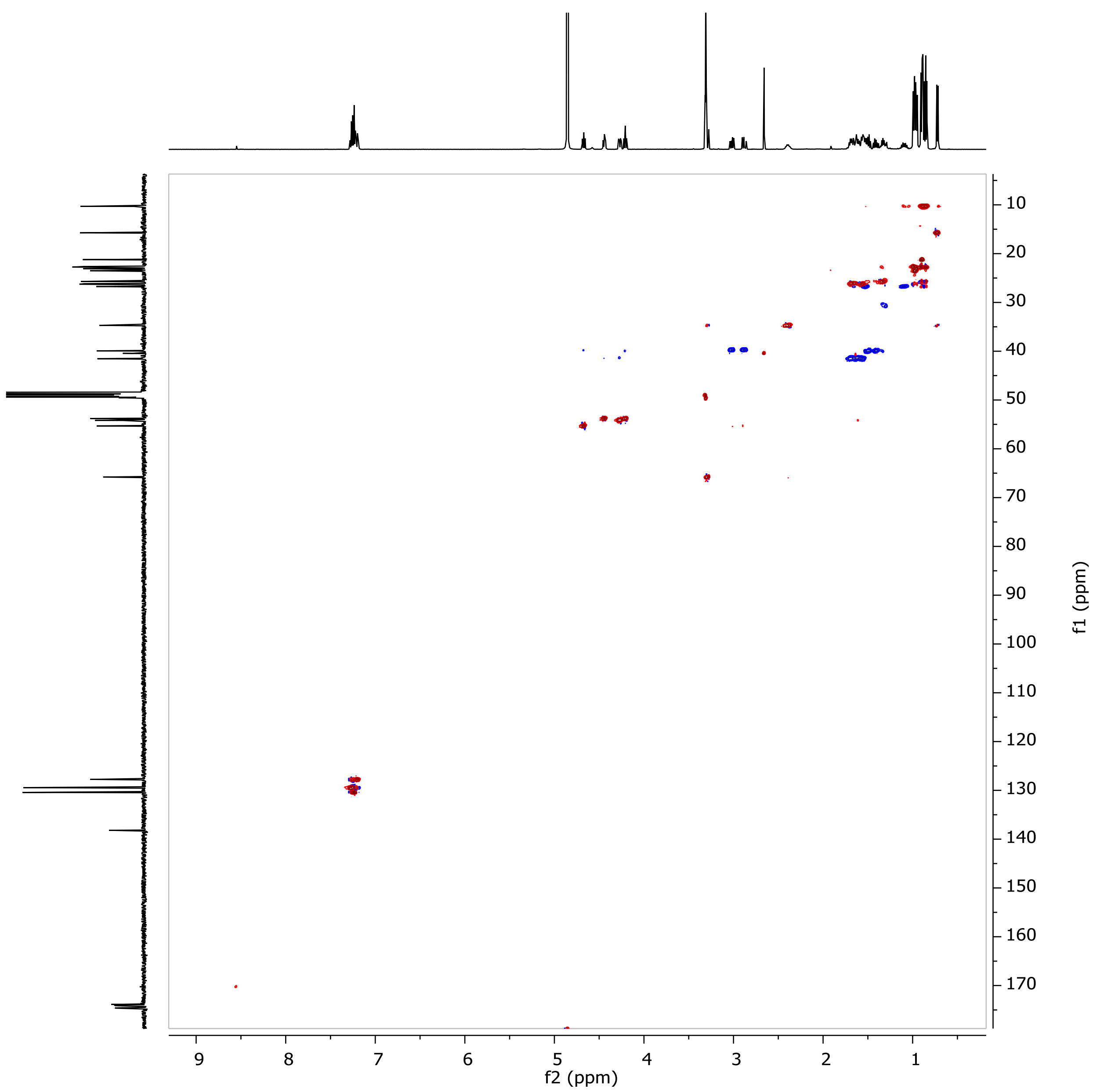

Figure S7. HSQC spectrum of **1** in methanol-d_4 at 500 MHz.

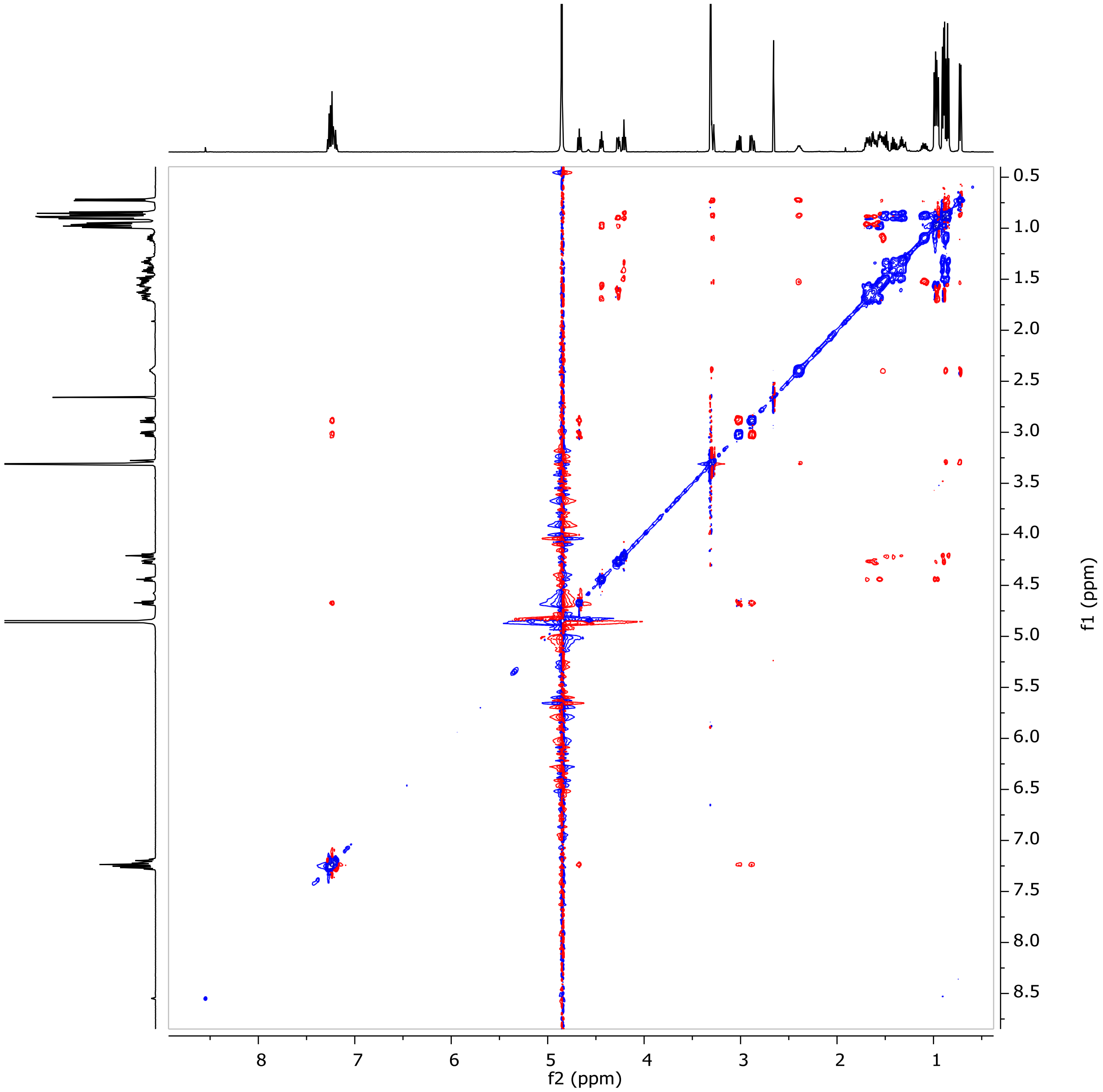

Figure S8. ROESY spectrum of **1** in methanol-d_4 at 500 MHz.

Table S1. ^{1}H and ^{13}C NMR data of **1** and *cyclo*-(L-Phe-L-Leu^{1}-L-Leu^{2}-L-Leu^{3}-L-Ile).

pos.	**1**		*cyclo*-(L-Phe-L-Leu^{1}-L-Leu^{2}-L-Leu^{3}-L-Ile)	
	δ_C,[a,c] type	δ_H[b] multi (*J*[Hz])	δ_C,[d] type	δ_H[e] multi (*J*[Hz])
Phe				
1	173.9, CO		171.6, CO	
2	55.3, CH	4.67 dd (8.4, 7.2)	52.7, CH	4.64 ddd (9.0, 8.5, 7.0)
3	39.8, CH_2	*α* 2.88 dd (13.4, 8.4)<br>*β* 3.02 dd (13.4, 7.3)	38.4, CH_2	*α* 2.73 dd (13.5, 9.0)<br>*β* 2.91 dd (13.5, 7.0)
4	138.2, C		137.3, C	
5,9	130.4, CH	7.24 d (7.0, 2H)	127.9, CH	7.18–7.23
6,8	129.4, CH	7.27 t (7.0, 2H)	129.0, CH	7.18–7.23
7	127.8, CH	7.20 t (7.0)	126.1, CH	7.18–7.23
NH	-	-	-	7.95 d (8.5)
Leu^{1}				
1	174.0, CO		171.4, CO	
2	53.79, CH	4.44 t (7.3)	51.8, CH	4.17 ddd (7.5, 7.5, 6.5)
3	41.5, CH_2	*α* 1.56 m *β* 1.70 m	38.6, CH_2	1.38 m
4	26.3, CH	1.56 (overlapped)	24.0, CH	1.33 m
5	21.2, CH_3	0.89 d (6.0)	22.2, CH_3	0.77 d (6.0)
6	23.5, CH_3	0.97 d (6.0)	22.3, CH_3	0.84 d (6.0)
NH	-	-	-	8.58 d (6.5)
Leu^{2}				
1	174.58, CO		171.5, CO	
2	54.2, CH	4.27 dd (10.4, 4.7)	51.9, CH	4.08 ddd (9.5, 8.0, 5.5)
3	41.4, CH_2	*α* 1.57 *β* 1.64 m	39.9, CH_2	1.51 m
4	26.2, CH	1.67 m (overlapped)	24.3, CH	1.60 m
5	23.1, CH_3	0.95 d (6.1)	20.7, CH_3	0.79 d (6.0)
6	22.6, CH_3	0.99 d (6.1)	23.0, CH_3	0.88 d (6.0)
NH	-	-	-	8.46 d (8.0)
Leu^{3}				
1	174.64, CO		171.1, CO	
2	53.83, CH	4.21 t (7.8)	52.1, CH	4.30 ddd (7.5, 7.5, 7.5)
3	39.9, CH_2	*α* 1.42 m *β* 1.49 m	40.2, CH_2	1.55 m
4	25.7, CH	1.33 (overlapped)	24.5, CH	1.46 m
5	22.79, CH_3	0.85 (d, 6.5)	22.5, CH_3	0.88 (d, 6.0)
6	22.76, CH_3	0.90 (d, 6.5)	22.2, CH_3	0.91 (d, 6.0)
NH	-	-	-	7.23 d (7.5)
Ile				
1	174.1, CO		171.0, CO	
2	65.8, CH	3.29 d (13.2)	62.8, CH	3.28 dd (8.5, 3.5)
3	34.7, CH	2.38 m	33.0, CH	2.23 m
4	26.7, CH	*α* 1.09 m *β* 1.53 m	25.0, CH	*α* 1.01 m *β* 1.41 m
5	10.3, CH_3	0.87 t (7.5)	9.8, CH_3	0.78 t (7.5)
6	15.7, CH_3	0.72 d (6.8)	15.1, CH_3	0.63 d (6.5)
NH	-	-	-	8.33 d (8.0)

Measured in methanol-d_4 at [a] 150 MHz for ^{13}C and at [b] 600 MHz for ^{1}H. [c] Assignment confirmed by HMBC and HSQC spectra. Measured in DMSO-d_6 at [d] 125 MHz for ^{13}C and at [e] 500 MHz for ^{1}H.

Display Report

Analysis Info

Acquisition Date 17.07.2024 01:02:35

Analysis Name	S:\DATA\AmaZon\swo23-Bank Wongkanoun\2024\Esteban\XY01542\XY01542_F5_RICE_Ethyl_Acetate_GB5_01_17774.d		
Method	17774.m	Operator	lab
Sample Name	XY01355_F5_RICE_Ethyl_Acetate	Instrument	amaZon speed
Comment			

Acquisition Parameter

Ion Source Type	ESI	Ion Polarity	Negative	Alternating Ion Polarity	on
Mass Range Mode	UltraScan	Scan Begin	100 m/z	Scan End	2000 m/z
Accumulation Time	2271 µs	RF Level	100 %	Trap Drive	68.9
SPS Target Mass	1000 m/z	Averages	6 Spectra		

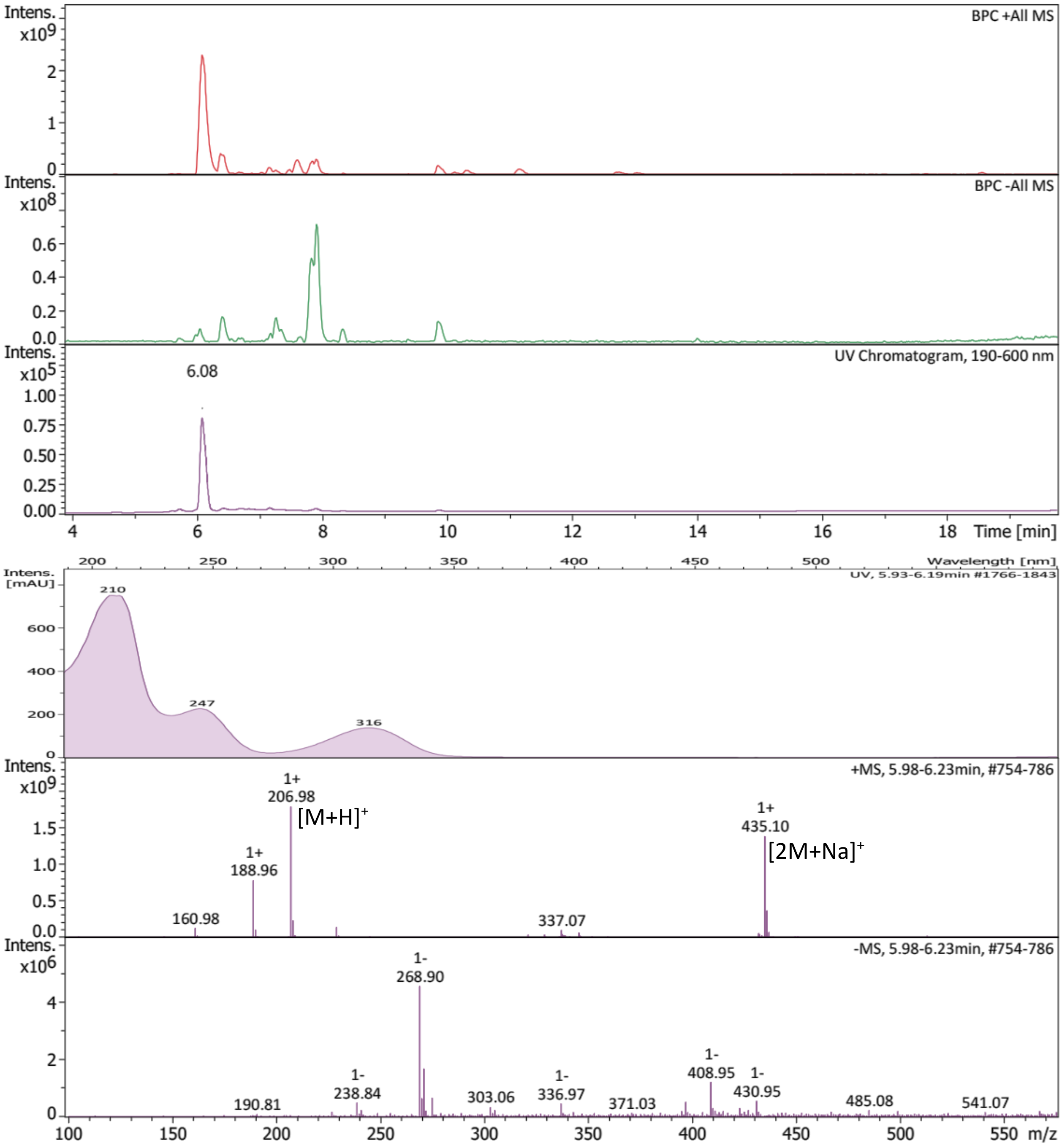

Bruker Compass DataAnalysis 6.1 printed: 12.09.2024 06:43:45 by: sel22 Page 1 of 1

Figure S9. LR-ESI-MS of **2**.

Display Report

Analysis Info

		Acquisition Date	08.08.2024 13:35:04
Analysis Name	S:\DATA\timsTOF\SWO24_Bank_SarunyouWongkanoun\24_08\XY01542-F5_P1-B-1_1_4011.d		
Method	MWIS-4D-Metabolomics_mz85-1500.m	Operator	Admin
Sample Name	XY01542-F5	Instrument	timsTOF Pro 2
Comment			

Acquisition Parameter

Ion Polarity Positive

SPS Target Mass

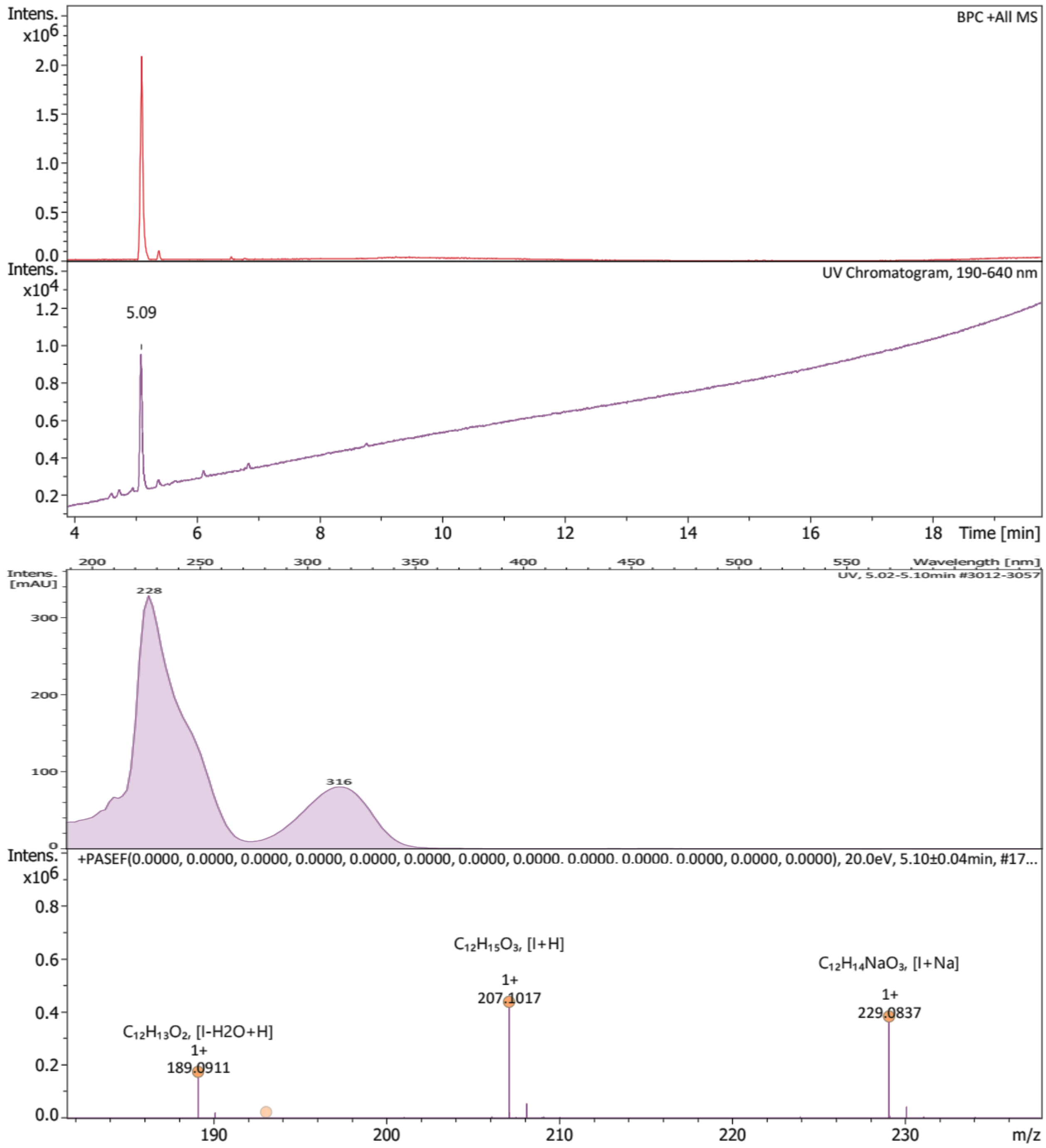

Bruker Compass DataAnalysis 6.1 printed: 12.09.2024 06:39:37 by: sel22 Page 1 of 1

Figure S10. HR-ESI-MS of **2**.

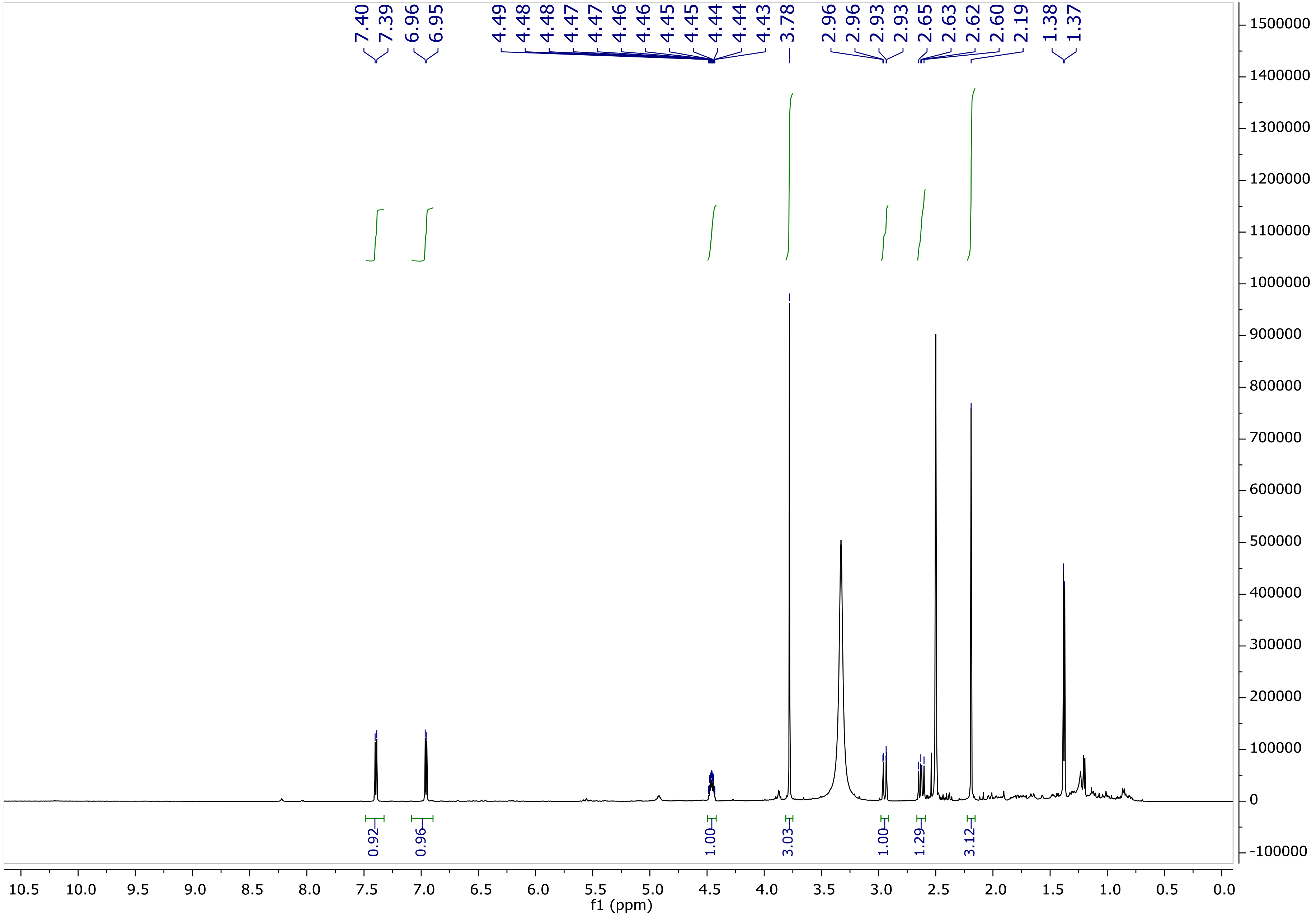

Figure S11. ^{1}H NMR spectrum of **2** in DMSO-d_6 at 500 MHz.

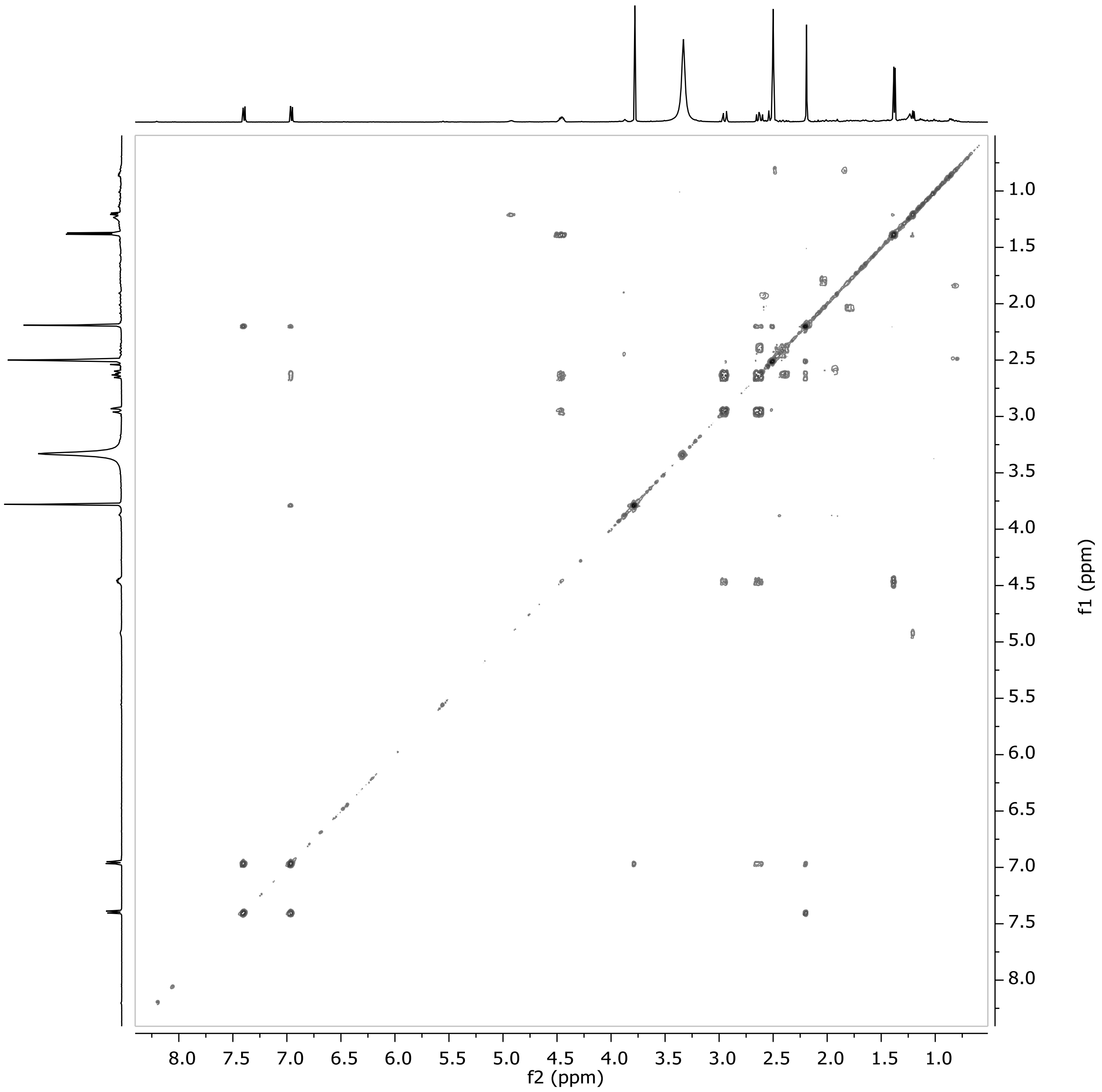

Figure S12. ^{1}H-^{1}H COSY spectrum of **2** in DMSO-d_6 at 500 MHz.

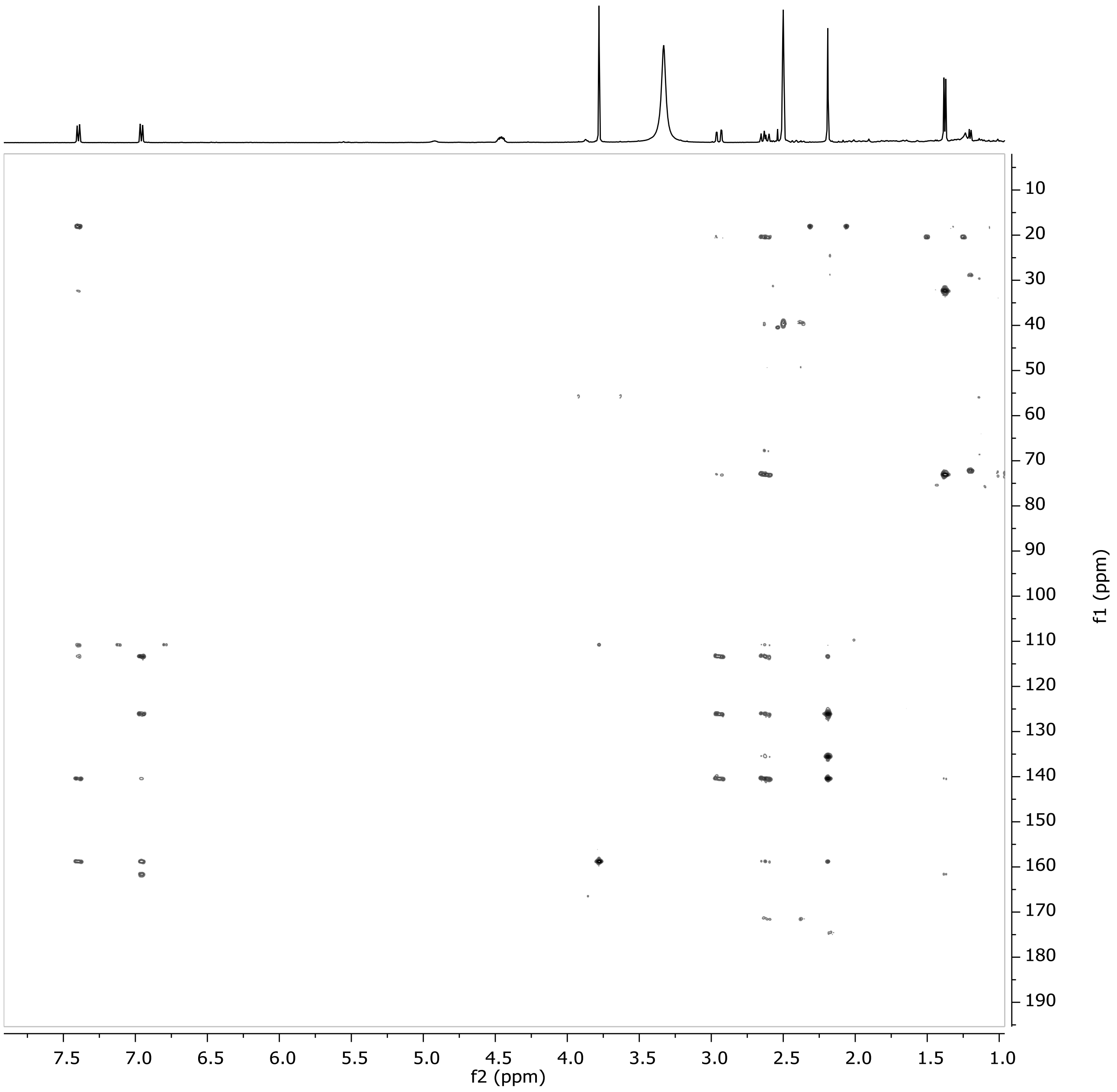

Figure S13. HMBC spectrum of **2** in DMSO-d_6 at 500 MHz.

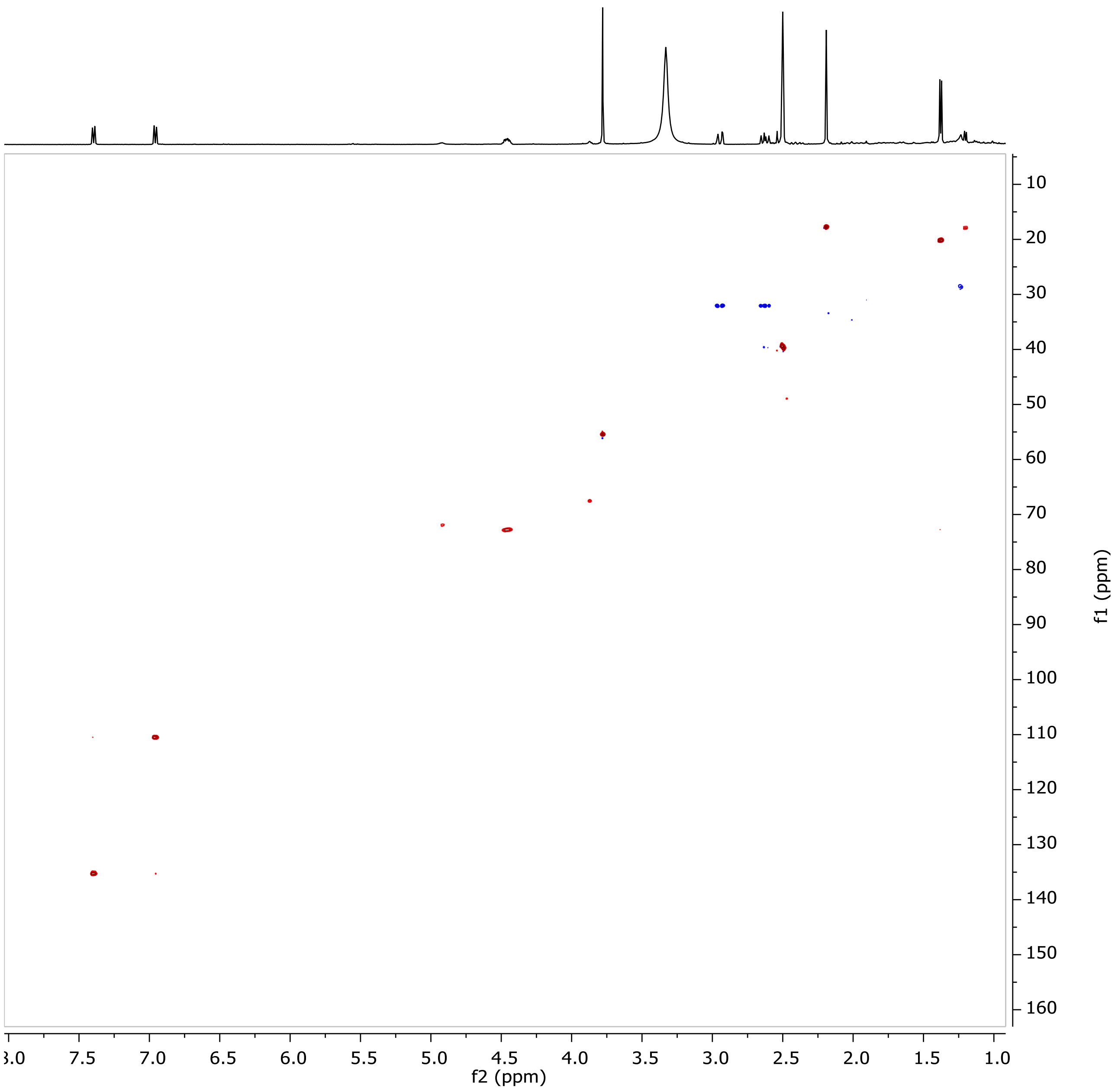

Figure S14. HSQC spectrum of **2** in DMSO-d_6 at 500 MHz.

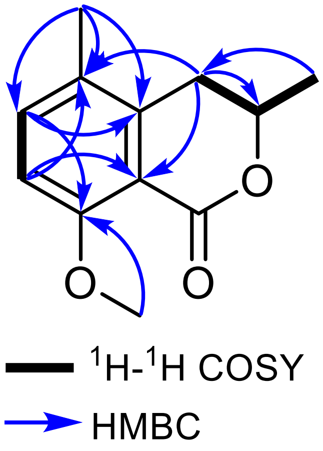

Figure S15. Key ^{1}H-^{1}H COSY and HMBC correlations of **2**.

Table S2. ^{1}H and ^{13}C NMR data of **2** and 3,5-dimethyl-8-hydroxy-7-methoxy-3,4-dihydroisocoumarin.

pos.	**2**		3,5-Dimethyl-8-hydroxy-7-methoxy-3,4-dihydroisocoumarin
	δ_C,[a,b] type	δ_H[a] multi (*J*[Hz])	δ_H[c] multi (*J*[Hz])
1	161.5, CO		
3	72.8, CH	4.46 dqd (11.4, 6.4, 2.8)	4.50 m
4	32.1, CH_2	*α* 2.95 dd (16.5, 2.8)<br>*β* 2.63 dd (16.5, 11.4)	*α* 2.87 dd (16.4, 2.9)<br>*β* 2.68 dd (16.4, 11.4)
4a	125.9, C		
5	140.3, C		
6	135.3, CH	7.40 d (8.6)	7.31 d (8.6)
7	110.6, CH	6.96 d (8.6)	6.83 d (8.6)
8	158.6, C		
8a	113.2, C		
9	20.2, CH_3	1.38 d (6.4)	1.50 d (6.3)
10	17.8, CH_3	2.19 s	2.23 s
11	55.5, CH_3	3.78 s	3.92 s

Measured in methanol-d_4 at [b] 600 MHz for ^{1}H. [b] Assignment based on HMBC and HSQC spectra.
[c] Measured in chloroform-*d* at 400 MHz for ^{1}H.

Table S3. Cytotoxicity (IC_{50}) and antimicrobial activity (MIC) of **1** and **2**.

**Test Cell Line**	**IC_{50} (µM)**	**Selectivity index**	**Positive Control (nM)**
	**1**		
Mouse fibroblast (L929; ACC 2)	>61.8	--	0.65[E]
Human endocervical adenocarcinoma (KB3.1; ACC 158)	5.2	11.9	0.17[E]
Human prostate carcinoma (PC-3; ACC 465)	10.7	5.8	0.09[E]
Human breast adenocarcinoma (MCF-7; ACC 115)	5.5	11.2	0.07[E]
Human lung carcinoma (A549; ACC 107)	4.7	13.1	0.05[E]
**Test Microorganism**	**MIC (µg/mL)**		**Positive Control (µg/mL)**
Staphylococcus aureus (DSM 346)	>66.6		0.42[G]
Escherichia coli (DSM 1116)	>66.6		0.83[G]
Bacillus subtilis (DSM 10)	>66.6		16.6[O]
Pseudomonas aeruginosa (PA 14)	>66.6		0.42[G]
Acinetobacter baumannii (DSM 30008)	>66.6		1.04[C]
Chromobacterium violaceum (DSM 30191)	>66.6		1.67[G]
Mycobacterium smegmatis (ATCC 700084)	>66.6		1.70[K]
Mucor hiemalis (DSM 2656)	>66.6		8.30[N]
Candida albicans (DSM 1665)	>66.6		8.30[N]
Rhodotorula glutinis (DSM 10134)	>66.6		4.20[N]
Schizosaccharomyces pombe (DSM 70572)	>66.6		8.30[N]
Wickerhamomyces anomalus (DSM 6766)	>66.6		8.30[N]

n.t.: Not tested.

E: Epothilone; T: Taxol; G: Gentamicin; O: Oxytetracycline; N: Nystatin; C: Ciprofloxacin; K: Kanamycin.
